# Open Culture Ethanol-Based Chain Elongation to Form Medium Chain Branched Carboxylates and Alcohols

**DOI:** 10.3389/fbioe.2021.697439

**Published:** 2021-08-17

**Authors:** Kasper D. de Leeuw, Theresa Ahrens, Cees J. N. Buisman, David P. B. T. B. Strik

**Affiliations:** Environmental Technology, Wageningen University & Research, Wageningen, Netherlands

**Keywords:** microbial chain elongation, open culture fermentation, branched carboxylates, medium chain fatty acids, medium chain fatty alcohols, bioprocess engineering

## Abstract

Chain elongation fermentation allows for the synthesis of biobased chemicals from complex organic residue streams. To expand the product spectrum of chain elongation technology and its application range we investigated 1) how to increase selectivity towards branched chain elongation and 2) whether alternative branched carboxylates such as branched valerates can be used as electron acceptors. Elongation of isobutyrate elongation towards 4-methyl-pentanoate was achieved with a selectivity of 27% (of total products, based on carbon atoms) in a continuous system that operated under CO_2_ and acetate limited conditions. Increasing the CO_2_ load led to more *in situ* acetate formation that increased overall chain elongation rate but decreased the selectivity of branched chain elongation. A part of this acetate formation was related to direct ethanol oxidation that seemed to be thermodynamically coupled to hydrogenotrophic carboxylate reduction to corresponding alcohols. Several alcohols including isobutanol and n-hexanol were formed. The microbiome from the continuous reactor was also able to form small amounts of 5-methyl-hexanoate likely from 3-methyl-butanoate and ethanol as substrate in batch experiments. The highest achieved concentration of isoheptanoate was 6.4 ± 0.9 mM Carbon, or 118 ± 17 mg/L, which contributed for 7% to the total amount of products (based on carbon atoms). The formation of isoheptanoate was dependent on the isoform of branched valerate. With 3-methyl-butanoate as substrate 5-methylhexanoate was formed, whereas a racemic mixture of L/D 2-methyl-butanoate did not lead to an elongated product. When isobutyrate and isovalerate were added simultaneously as substrates there was a large preference for elongation of isobutyrate over isovalerate. Overall, this work showed that chain elongation microbiomes can be further adapted with supplement of branched-electron acceptors towards the formation of iso-caproate and iso-heptanoate as well as that longer chain alcohol formation can be stimulated.

## Introduction

Chain elongation fermentation is an emerging bioprocess by which medium chain carboxylates (MCCs) can be produced. Currently, MCCs are mainly produced from either non-renewable fossil resources or palm and kernel oil-crops (Ahmad et al., 2019; Tan and Lim, 2019). Chain elongation fermentations provide a sustainable alternative by utilizing organic residue streams as substrate (Agler et al., 2011; Agler et al., 2012). In general, these fermentations require electron acceptors in the form of short chain carboxylates (e.g., acetate, propionate, butyrate, valerate), that can be obtained after hydrolysis and acidification of organic residues. An electron donor such as ethanol is then required to elongate the short chain carboxylates to MCCs (De Groof et al., 2019). Microbial chain elongation provides an economically attractive alternative to biogas formation (Kleerebezem et al., 2015). MCCs in general can be used in the production of e.g., solvents, feed additives (Liu et al., 2018), lubricants, surfactants, emulsifiers, pharmaceuticals (Angenent et al., 2016) and as precursors for plastics and fuels (Levy et al., 1984; Zhang et al., 2004). The amount of research on the formation of straight MCCs such as n-caproate (n-C_6_) and n-caprylate (n-C_8_) via microbial chain elongation is quickly expanding (Steinbusch et al., 2011; Zhu et al., 2015; Angenent et al., 2016; Zhu et al., 2017; Chwialkowska et al., 2019; Han et al., 2019; Leng et al., 2019). Chain elongation fermentation technology could become an impactful recycling method that can aid in the development of a circular economy (De Groof et al., 2019).

This research focuses on expanding the product spectrum of microbial chain elongation using synthetic media, with the aim to broaden the application range of the technology. Recently it was also shown that branched MCC isocaproate (i-C_6_) can be formed in considerable amounts when isobutyrate (i-C_4_) is used as electron acceptor in an open-culture ethanol based chain elongation fermentation (de Leeuw et al., 2019). This research suggests that formation of other branched MCCs such as isoheptanoate (i-C_7_) should be possible. The necessary isovalerate (i-C_5_) substrate could be formed prior to chain elongation via protein degradation steps during acidification (Allison, 1978; Yang et al., 2016). Branched MCCs exhibit different physical properties compared to straight MCCs, which can make them more suitable for various applications. Branched MCCs have a higher viscosity, a reduced crystallization temperature (Lee et al., 1995) and have an oxidative stability (Zhang et al., 2004; Atsumi et al., 2008) that can improve fuel combustion (Ghosh et al., 2006).

In addition to MCCs, higher alcohols can be coproduced within a chain elongation microbiome (Diender et al., 2016; Richter et al., 2016; de Leeuw et al., 2019). In some cases, acetate and CO_2_ limitation triggered alcohol formation during ethanol-based chain elongation (de Leeuw et al., 2019). During syngas-facilitated chain elongation a low pH (∼4.8) triggered alcohol formation (Ganigué et al., 2016). A better understanding on their production mechanism could facilitate the development of higher alcohol formation from complex organic residues, rather than from more expensive glucose-based fermentations (Chen et al., 2013; Xin et al., 2014).

In open culture fermentations the microbiomes should be enriched in such a way that the metabolic fluxes responsible for electron donor and acceptor consumption are directed towards the formation of the desired products. A challenge with ethanol-based chain elongation fermentations is minimizing the activity of direct ethanol oxidation, that can occur independent of chain elongation and is directly competing for the electron donor ethanol. For ethanol-based chain elongation the following stoichiometry is observed (Angenent et al., 2016):

$$5C_{X}H_{\mathrm{2X-1}}O_{2}^{-}+6C_{2}H_{6}O\to 5C_{\mathrm{X+2}}H_{\mathrm{2X+3}}O_{2}^{\mathrm{-1}}+C_{2}C_{3}O_{2}^{-}+4H_{2}\mathrm{O+}H^{+}+2H_{2}$$ (equation 1)

With (excessive) direct ethanol oxidation this following conversion is regarded (Roghair et al., 2018a):

$$C_{2}H_{6}\mathrm{O+}H_{2}O\to C_{2}H_{3}O_{2}^{-}+H^{+}+2H_{2}$$ (equation 2)

Direct ethanol oxidation is thermodynamically feasible at a hydrogen partial pressure below approximately 1 kPa and can be stimulated when syntrophic partners in biofilms utilize the produced hydrogen (Seitz et al., 1990; Nozhevnikova et al., 2020). The usage of ethanol has a big impact on the sustainability and costs of the ethanol-based chain elongation process, and as such excess consumption should be avoided at all cost (Chen et al., 2017). Additionally, the *in-situ* acetate formation reduces the selectivity of branched and odd-chain carboxylate elongation (Grootscholten et al., 2013a; de Leeuw et al., 2019). Earlier research has shown that reducing the CO_2_ dosage could reduce excessive ethanol oxidation and increase carboxylate elongation selectivity (Roghair et al., 2018a).

The objective of this study was to investigate branched electron acceptors for the formation of i-C_6_ and i-C_7_ by ethanol-based chain elongation reactor microbiomes. A continuous anaerobic filter bioreactor that was fed with ethanol and i-C_4_ was operated under two different CO_2_ loads and used enrich a microbiome for chain elongation activity. The results indicate that branched chain elongation selectivity was indeed higher at low CO_2_ loads. This effect, however, could be transient due to microbiome adaptation that led to increased functionality of alcohol formation, which seemed to be coupled to direct ethanol oxidation.

The same microbiome was used in batch experiments to evaluate the feasibility of using branched five-carbon carboxylates (i-C_5_ and L/D 2-methyl butanoate) as substrate and electron acceptor within an ethanol-based chain elongation microbiome. Hypothetically L/D 4-methylhexanoate is the elongation product of L/D 2-methylbutyrate, whereas i-C_7_ is the elongation product of i-C_5_, assuming the elongation occurs in a similar fashion as during earlier observed i-C_4_ elongation to i-C_6_ and other chain elongation mechanisms (Kim et al., 2019). We show the likely formation of i-C_7_ via microbial i-C_5_ (3-methylbutanoate) elongation using ethanol as electron donor. After this observation, another batch series was performed to evaluate the effect of higher initial hydrogen partial pressure and acetate concentrations that are known to influence the chain elongation activity (Steinbusch et al., 2011; Spirito et al., 2014; Angenent et al., 2016; de Leeuw et al., 2019). In addition, substrate specificity of i-C_5_ was compared to that of i-C_4_ as an alternative substrate and electron acceptor. The results highlight that branched chain elongation hypothetically occurs as cometabolism during straight chain elongation, meaning that a minimum amount of acetate is required for branched chain elongation to occur at all.

## Materials and Methods

### Continuous Reactor Setup

A continuous anaerobic filter bioreactor was set up to investigate the effect of CO_2_ supply on isobutyrate chain elongation. The reactor system (35 cm height, an internal column diameter of 6.5 cm, a 1 L working liquid volume, and a headspace of 0.15 L) was exactly the same as in previous research on isocaproate formation via ethanol based chain elongation (de Leeuw et al., 2019), except for the addition of a carrier material. This was done to retain microbial biomass and to increase the rate of chain elongation activity (Grootscholten et al., 2013b). After startup (phase I), the reactor was filled with sponge carrier material (0.15 L liquid exclusion volume of 15 by 15 mm polyurethane cubes; Recticel, Belgium) to support additional growth of biomass (phase II). To maintain anaerobic conditions during this procedure the reactor was flushed with N_2_ gas. The addition of cubes changed the active liquid volume of the reactor from 1 to 0.85 L. The influent rate was adjusted accordingly (from 22.2 mlh^−1^–18.9 mlh^−1^) to maintain a hydraulic retention time (HRT) of around 45 h. The CO_2_ supply was doubled in phase III and halved again in phase IV. An overview of the influent carbon sources, the steady state duration for each phase, the HRT, pH and the CO2 supply for the different phases are listed in Table 1.

**TABLE 1:** Overview over the different phases in the reactor. The influent carbon sources, the steady state duration for each phase, the HRT, pH and CO2 supply are listed.

Phase	I	II	III	IV
Condition	Start-up	Add carrier material	CO_2_ increase	CO_2_ decrease
Phase period (days)	1–45	45–78	78–94	94–129
HRT (h)	44 ± 7	46 ± 8	44 ± 2	47 ± 6
Isobutyrate (mM C)	650	650	650	650
Ethanol (mM C)	540	540	540	540
PH	6.65 ± 0.07	6.6 ± 0.03	6.6 ± 0.02	6.62
CO_2_ supply (NmL/min)	0.18	0.18	0.36	0.18

### Medium

The reactor was fed with 650 mmol Carbon L^−1^ (mMC) isobutyrate, 540 mMC ethanol and 1 gL^−1^ yeast extract [34 mMC (Duboc et al., 1995)] as carbon sources (acetate was omitted from the influent). The reactor and batch experiments were all done using the same macro- and micronutrient composition (g L^−1^): NH_4_H_2_PO_4_ 3.60; MgCl_2_·6H_2_O 0.33; MgSO_4_·7H_2_O 0.20; CaCl_2_·2H_2_O 0.20; KCl 0.15. In addition, the micronutrients (Pfennig trace metals and B-vitamins) of the designed basal medium described in Phillips j et al. (1993) was used.

### Batch Experimental Setup

The batch experiments were performed in 250 ml serum bottles (150 ml liquid medium). The remaining 100 ml gas headspace was replaced at the start of each batch up to a pressure of 150 kPa (see gas composition per batch in Tables 1–3). The batch bottles were kept in a shaker at 35°C and 150 rpm throughout the whole experiment. The exact step-by-step protocol for the batches is given in the Supplementary Information, including the recipes for the medium preparation stock solutions (Supplementary Tables S1, S4). All batches were carried out in duplicates.

### Investigating i-C5 Elongation Proof of Concept—First Batch Series

The first experimental series consisted of eight batches (1.A to 1.H) that aimed to investigate if an enriched microbiome that produced i-C_6_ could also elongate branched valerates to branched heptanoates. Ethanol (160 or 320 mMC) and acetate (6.5 or 13 mMC) were always added as substrate, whereas the types of branched valerates were varied throughout the series. In batch 1.A and 1.B a racemic mixture of L/D 2-methylbutanoate was added. I-C_5_ (i.e., 3-methylbutanoate) was added in batch 1.C and 1.D. In batch 1.E and 1.F a 50:50 mixture of the L/D 2-methylbutanoate racemate and i-C_5_ was added to investigate their combined effect on chain elongation. All these batches were performed at two different substrate concentrations (Table 2), because it was unknown to what degree the (potentially toxic) branched valerates could affect the chain elongation process. BES (2-bromoethanesulfanoate) was added at 10 g/L to inhibit methanogenesis (Vogels et al., 1982), except in the control batch 1.G. Additionally a control batch (1.H) was performed without yeast extract to be able to exclude the possible formation of i-C_7_ from yeast extract.

**TABLE 2:** Overview of the different starting parameters (t = 0) for the first batch series, as well as final values (t = end) for product selectivites (% of total formed compounds based on carbon atoms) of i-C_7_, n-C_6_ and hexanol, the percentages of unconverted ethanol at the end of the batch, pH and partial pressures of CO_2_ and H_2_.

	1.A	1.B	1.C	1.D	1.E	1.F	1.G	1.H
EtOH (mM C)	320	160	320	160	320	160	160	160
Acetate (mM C)	13	6.5	13	6.5	13	6.5	6.5	6.5
L/D 2-methylbutanoate (mM C)	325	162.5	−	−	162.5	81.3	−	−
(3-) i-C_5_ (mM C)	−	−	325	162.5	162.5	81.3	162.5	162.5
BES (g/L)	10	10	10	10	10	10	−	10
Yeast (g/L)	0.5	0.5	0.5	0.5	0.5	0.5	0.5	−
pH	6.5	6.5	6.5	6.5	6.5	6.5	6.5	6.5
N_2_ (%)	90	90	90	90	90	90	90	90
CO_2_ (%)	10	10	10	10	10	10	10	10
H_2_ (%)	0	0	0	0	0	0	0	0
i-C_7_ selectivity (%)	0	0	4.1	2.9	0.6	0.9	5.4	2.5
n-C_6_ selectivity (%)	75.9	82.0	87.1	75.0	79.3	82.8	85.7	89.8
Hexanol selectivity (%)	1.5	3.5	4.3	6.0	2.8	3.8	2.9	4.5
Unconverted ethanol (%)	73.4	56.4	74.5	54.8	77.6	53.2	65.7	47.6
Final pH	5.9	5.9	6.1	5.8	6.0	5.9	5.7	6.1
Final CO_2_ partial pressure (kPa)	4.3	5.3	7.1	4.1	7.1	3.1	0.1	5.1
Final H_2_ partial pressure (kPa)	20.3	17.7	29.7	11.8	27.4	17.1	2.9	21.3

### Investigating the Substrate Specificity and Limiting Factors for Chain Elongation Activity—Second and Third Batch Series

In the first batch series ethanol was not completely converted and it remained unclear if this was caused by the drop in pH, a limiting acetate concentration, the increased hydrogen partial pressure or something else [such as product inhibition on the microbiome (Roghair et al., 2018b)]. Therefore, a second and third series were performed to further investigate the effect of increased hydrogen partial pressure in combination with different starting acetate concentrations. In contrast to the first series that contained no hydrogen at the start of the experiment, the second series was performed with hydrogen already present in the headspace at the start of the batch (20% for all batches, except 2.C which contained 80% H_2_ at the start). This was done to minimize acetate formation via potential excessive ethanol oxidation which is thermodynamically inhibited at higher H_2_ pressures (Roghair et al., 2018a) and to investigate the effect of an elevated H_2_ pressure on the chain elongation itself (Li et al., 2008; Angenent et al., 2016). These batches were all started at pH 7 instead of 6.5 to allow for more proton formation due to ethanol oxidation before pH drops down to limiting levels. When the pH drops below 6.3 it could cause limitations in bicarbonate availability (Tomlinson, 1954; Jungermann et al., 1968). When the pH drops even further and approaches pK values of the carboxylates (∼4.9), undissociated fatty acids concentrations rise which causes additional toxicity effects (Infantes et al., 2012). One batch (2.B) was started with an initial acetate concentration ten times higher than the control (2.A). Additionally, to investigate the necessity of acetate during chain elongation, batch 3.B was started with zero added acetate (3.A as control, in the third batch series).

To batch 2.D i-C_4_ was added in addition to i-C_5_ to get insight into substrate preferences for branched chain elongation. In the third batch series n-valerate was added (batch 1.D) to compare its utilization as electron acceptor with i-C_5_ and exclude possible i-C_7_ formation via n-C_5_. Tables 3, 4 show overviews of the second and third batch series, respectively. The medium was the same as the medium from the first series, except for the indicated changes in the tables.

**TABLE 3:** Overview of the different starting parameters (t = 0) for the second batch series, as well as final values (t = end) for product selectivites (% of total formed compounds based on carbon atoms) of i-C_7_, n-C_6_ and hexanol, the percentages of unconverted ethanol at the end of the batch, pH and partial pressures of CO_2_ and H_2_.

	2.A (low acetate)	2.B (high acetate)	2.C (high hydrogen)	2.D (including i-C_4_)
Inoculum	Batch 1.D	Batch 1.D	Batch 1.D	Batch 1.D
EtOH (mM C)	160	160	160	160
(3-) i-C_5_ (mM C)	162.5	162.5	162.5	162.5
i-C_4_ (mM C)	−	−	−	64.5
Acetate (mM C)	6.5	65	6.5	6.5
BES (g/L)	10	10	10	10
Yeast (g/L)	0.5	0.5	0.5	0.5
pH	7	7	7	7
N_2_ (%)	70	70	10	70
CO_2_%	10	10	10	10
H_2_ (%)	20	20	80	20
i-C_7_ selectivity (%)	7.3	1.4	7.1	4.2
n-C_6_ selectivity (%)	79.4	81.9	80.8	56.9
Hexanol selectivity (%)	4.8	2.1	3.9	1.4
Unconverted ethanol (%)	58.0	4.8	72.5	61.4
Final pH	6.5	6.3	6.6	6.6
Final CO_2_ partial pressure (kPa)	1.8	2.1	2.2	3.8
Final H_2_ partial pressure (kPa)	32.1	60.0	110.7	41.9

**TABLE 4:** Overview of the different starting parameters (t = 0) for the third batch series, as well as final values (t = end) for product selectivites (% of total formed compounds based on carbon atoms) of i-C_7_, n-C_6_ and hexanol, the percentages of unconverted ethanol at the end of the batch, pH and partial pressures of CO_2_ and H_2_.

	3.A (low acetate)	3.B (no acetate)	3.C (no extra carboxylate)	3.D (n-C_5_)
Inoculum	regrown 2.D	regrown 2.D	regrown 2.D	regrown 2.D
EtOH (mM C)	320	320	320	320
(3-) i-C_5_ (mM C)	162.5	162.5	—	—
n-C_5_ (mM C)	—	—	—	162.5
Acetate (mM C)	13	0	13	13
BES (g/L)	10	10	10	10
Yeast (g/L)	0.5	0.5	0.5	0.5
pH	7	7	7	7
N_2_ (%)	60	60	60	60
CO_2_%	20	20	20	20
H_2_ (%)	20	20	20	20
i-C_7_ selectivity (%)	3.2	3.8	0	0
n-C_6_ selectivity (%)	83.2	82.3	68.2	62.5
Hexanol selectivity (%)	1.8	1.8	1.6	2.4
Unconverted ethanol (%)	53.1	58.0	22.1	41.6
Final pH	6.0	6.1	5.8	6.0
Final CO_2_ partial pressure (kPa)	0.3	0.4	0.3	1.1
Final H_2_ partial pressure (kPa)	15.0	8.9	10.2	18.9

### Inoculum

The continuous reactor as well as the first batch series was inoculated with a mixture of two anaerobic cultures. One volume part was taken from the continuous reactor that elongated i-C_4_ to i-C_6_ (de Leeuw et al., 2019) and contained a *C. kluyveri* dominant culture; second equal volume part came from an undefined mixed bovine rumen sample. The bovine rumen liquid from three cows was provided by the Animal Science Department of Wageningen University and Research. Biomass concentration was not measured within the inocula. The inocula were centrifuged at 4,500 rpm for 10 min after which the cell pellets were resuspended in carbon source free medium prior to inoculation as described within the step-by-step protocol in the Supplementary Information section.

The inoculum for the second batch series was taken from batch 1.D of the first series. Its contents were centrifuged in 50 ml tubes at 4,500 rpm and the pellets were subsequently combined and re-suspended with 50 ml carbon source free medium. These re-suspended cells were then used as inoculum for the second batch series as described within the step-by-step protocol. Similarly, the third batch series was inoculated with biomass that originated from batch 2.D. However, before inoculating, batch 2.D was stored for one and a half year at room temperature. Sporulation of bacteria was observed under the microscope, prior to activation. Before starting the third batch series an activation batch was performed using the same conditions as in batch 2.D. The third batch series was then inoculated with this freshly activated biomass.

### Sampling and Measurement

Samples of the gas phase were taken once per week and analyzed using an established protocol for gas chromatography to determine the fractions of O_2_, N_2_, CH_4_, H_2_, and CO_2_ (Steinbusch et al., 2011; Chen et al., 2016). Before sampling the pressure was measured using a pressure meter (GMH 3151). At the same time liquid samples (3.5 ml) were taken, centrifuged at 10,000 rpm and stored in a freezer at −20°C. Every 2 weeks these samples were analyzed according an earlier described gas chromatography method (Agilent 7890B, United States, HP-FFAP column, FID detector) (Jourdin et al., 2018) to determine the concentrations of primary alcohols and volatile carboxylic acids (ethanol, propanol, butanol, iso-butanol, pentanol, branched pentanols, n-hexanol, iso-hexanol and acetate, n-butyrate, iso-butyrate, n-valerate, branched valerates, n-caproate, iso-caproate, n-heptanoate, iso-heptanoate and n-caprylate). The branched valerates i-C_5_ and L/D 2-methylbutyrate as well as the branched pentanols isopentanol and L/D 2-methylbutanol could not be distinguished with the available equipment because the isomers exhibited the same retention time. Therefore, the batches were designed to investigate their effect on chain elongation separately to analyze which isomers of branched valerates were used for the formation of which branched heptanoate. The expected forms of branched heptanoates, L/D 4-methylhexanoate and i-C_7_, could be distinguished as is shown in Supplementary Figure S1 in the Supplementary Information. For the continuous experiment, the data is presented using the averaged values during each phase and a confidence interval (±) using an α of 0.1. For depiction of the batches, the duplicate results are averaged, and the error bars indicate the differences between each measurement within the duplicate. For the carbon and electron balance calculation (see SI page 5 for equations) yeast extract was assumed to be completely consumed.

## Results

### Continuous Reactor

#### Increasing and Subsequently Decreasing CO_2_ Loading Rate

**FIGURE 1:**
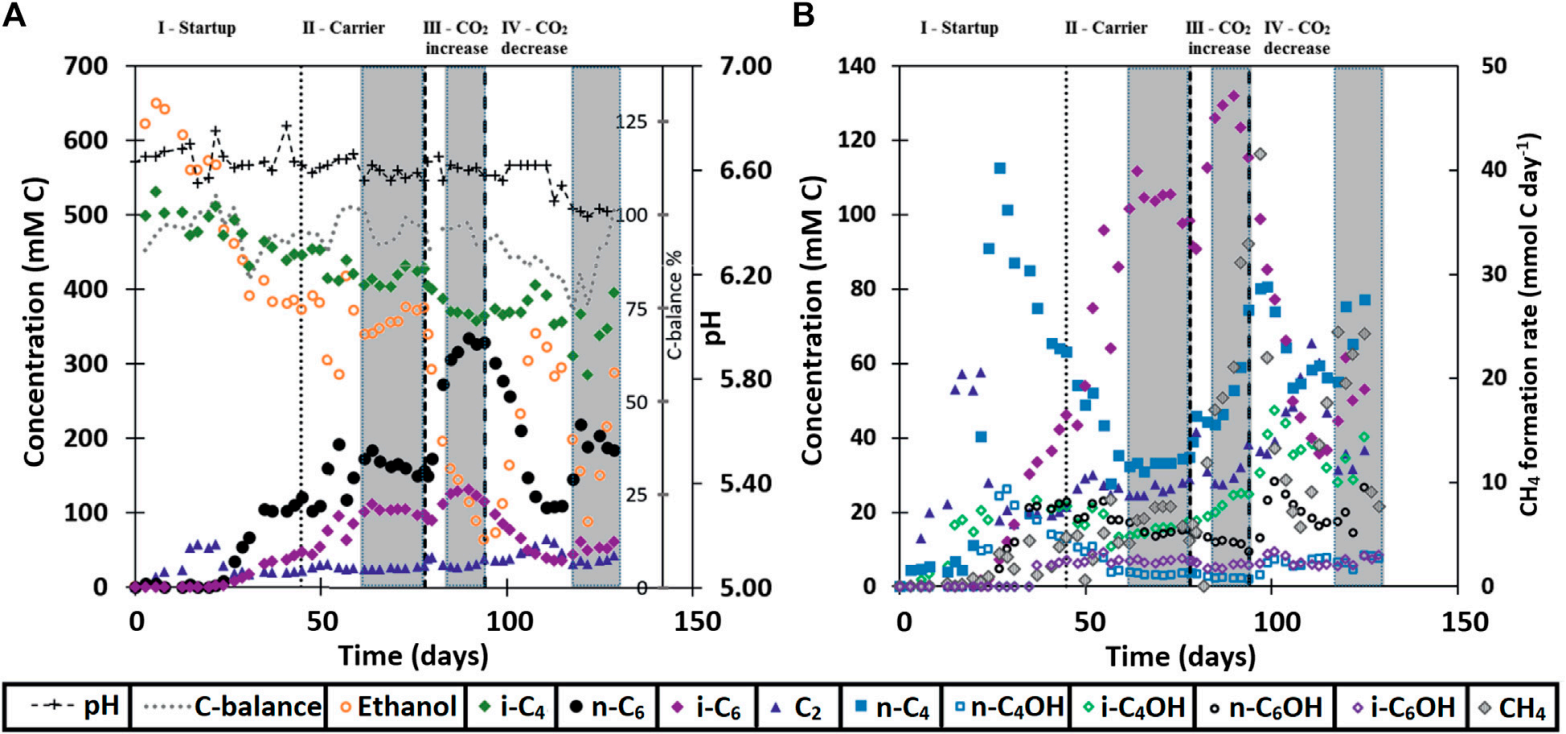
Broth concentrations of metabolites within the continuous reactor system. Additionally, the carbon balance and pH are shown in **(A)** and methane formation is rate shown in **(B)**. The grey boxes at the end of each phase show the range where the averages were taken for the values in Table 5.

During the first 3 weeks after starting the isobutyrate (i-C_4_) and ethanol (EtOH) fed continuous bioreactor, acetate (C_2_) accumulated in the broth, followed by n-butyrate (n-C_4_), n-butanol (n-C_4_OH) and isobutanol (i-C_4_OH) (Figure 1). Around day 30 the broth concentration of these compounds, except for i-C_4_OH, decreased while n-caproate (n-C_6_) and isocaproate (i-C_6_) formation started to occur. When n-C_6_ concentrations no longer increased at the end of the start-up (phase I) carrier material was added on day 45. The reactor then reached a steady state from day 62 to day 78 in phase II. The CO_2_ headspace partial pressure was consistently below 1 kPa as soon as chain elongation activity was observed, even after doubling the CO_2_ in phase III. The highest volumetric productivities and concentrations of i-C_6_ were obtained during phase III at increased CO_2_ supply, reaching a rate of 57 ± 4 mMC/day, or 1.1 ± 0.07 g/L/day and a concentration of 125 ± 6.6 mCM, or 2.43 ± 0.13 g/L (Table 5). However, selectivity towards i-C_6_ (carbon per tot carbon in products) was highest in phase II (27%) and dropped to 20% in phase III.

**TABLE 5:** Overview of averaged operating parameters and conversion rates including confidence intervals. Averages from phase IV are taken when caproate formation had stabilized (day 106–113).

	Phase II		Phase III		Phase IV	
Discription	Add carrier material		CO_2_ increase		CO_2_ decrease	
Phase period	day 45–78		day 78–94		day 94–129	
Averages taken from	day 62–78		day 85–94		day 117–129	
Calculated Excessive ethanol Oxidation (%)	21	±7	25	±6	44	±11
HRT (hours)	46	±8	44	±2	43	±1
pH	6.60	±0.03	6.60	±0.02	6.46	±0.04
CO_2_ loading (NmL min^−1^)	0.18		0.36		0.18	
C-balance (%)	96	±3	95	±3	87	±10
e-balance (%)	96	±3	97	±3	84	±9
Volumetric conversion rates (mmol C L^-1^ day^−1^)						
C_2_	13	±1	14	±2	17	±2
i-C_4_	−49	±8	−58	±6	−67	±20
n-C_4_	17	±0	25	±5	31	±4
i-C_6_	50	±3	57	±4	25	±3
n-C_6_	82	±5	146	±4	88	±11
n-C_8_	2	±0	5	±0	2	±0
EtOH	−134	±13	−219	±20	−203	±32
n-C_4_OH	2	±0	1	±0	3	±1
i-C_4_OH	8	±1	11	±1	16	±2
n-C_6_OH	8	±1	5	±1	10	±2
i-C_6_OH	1	±0	1	±0	1	±0
CH_4_ (gas)	6	±1	24	±8	18	±7
CO_2_ (gas)	−11	±0	−23	±0	−11	±0

Excessive ethanol oxidation as shown in the table is calculated from the observed chain elongation activity and ethanol consumption using an earlier described method (de Leeuw et al., 2019). It is a stoichiometric analysis by which the amount of ethanol consumption is evaluated versus the observed chain elongation activity, using a fixed stoichiometry for reverse β-oxidation (Eq. 1 in introduction) with

$$\mathrm{Excessive}\;\mathrm{ethanol}\;\mathrm{oxidation}\left(\%\right)=\left(1-\frac{\sum \mathrm{Chain}\;\mathrm{elongation}\;\mathrm{activty}}{\mathrm{observed}\;\mathrm{ethanol}\;\mathrm{consumption}}\right)$$ (equation 3)

where ∑ chain elongation activity is the amount of ethanol that is necessary to perform all observed chain elongation (Roghair et al., 2018c). This value gives an indication of how much ethanol is not used for chain elongation but is instead oxidized to acetic acid and hydrogen. The results in Table 5 show that excessive ethanol oxidation had increased from 21 ± 7% in phase II to 44 ± 11% in phase IV.

The reactor was not allowed to develop a steady state in phase III because methane formation kept increasing, which was deemed unfavorable for chain elongation activity in the long term. Instead, the CO_2_ load was lowered from 0.36 NmL/min (phase III) to 0.18 NmL/min (phase IV) with two aims: 1) lower methanogenic activity and 2) investigate if the i-C_6_ selectivity could be increased again. However, the reactor had also developed an increased alcohol (in particular i-C_4_OH) productivity (see Table 5). Consequently, different conversion rates were observed in phase VI compared to phase II, although reactor operating conditions were the same. Alcohol (n-C_4_OH, i-C_4_OH, n-C_6_OH and i-C_6_OH) formation had increased from a combined selectivity of 10% in phase II to 16% in phase IV. Hexanol and isobutanol were the most predominant higher alcohols at concentrations of 19 ± 3 mMC (327 ± 47 mgL^−1^) and 37 ± 1 mMC (684 ± 24 mgL^−1^), respectively. Also, an increase in straight carboxylates was observed relative to phase II, while i-C_6_ selectivity had dropped down to 12% in phase IV. Additionally, the hydrogen partial pressure had dropped below 1 kPa from phase III onwards and did not recover to the levels observed in phase II (up to 10 kPa). The gas partial pressures in the reactor headspace are shown in Supplementary Figure S2 in the SI. Evidently, the average conversion rates in the reactor show a non-reversible behavior after the CO_2_ increase and decrease.

### Batches

#### Chain Elongation With Alternative Electron Acceptors During Acetate Limitation

**FIGURE 2:**
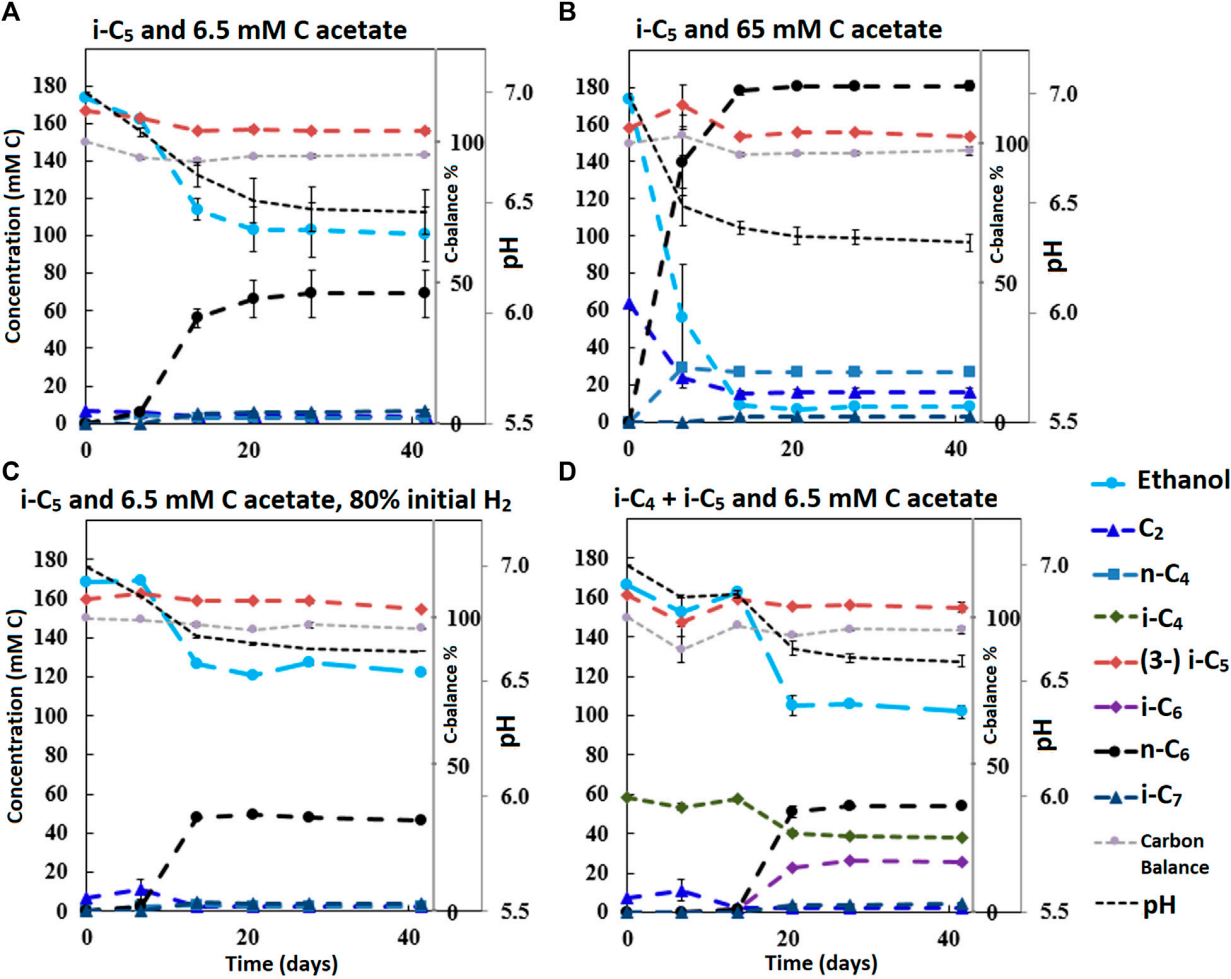
Concentration profiles of batches performed with i-C_5_ as additional electron donor and varying levels of starting acetate amounts and hydrogen partial pressures **(A–C)**. Batch **(D)** shows the preference for i-C_4_ over i-C_5_ as alternative electron acceptor leading to branched MCFA formation concomitant with straight chain elongation. The i-C_5_ addition results only in marginal i-C_7_ formation. Concentration profiles of components that were present in lower concentration ranges are shown in Supplementary Figure S7.

**FIGURE 3:**
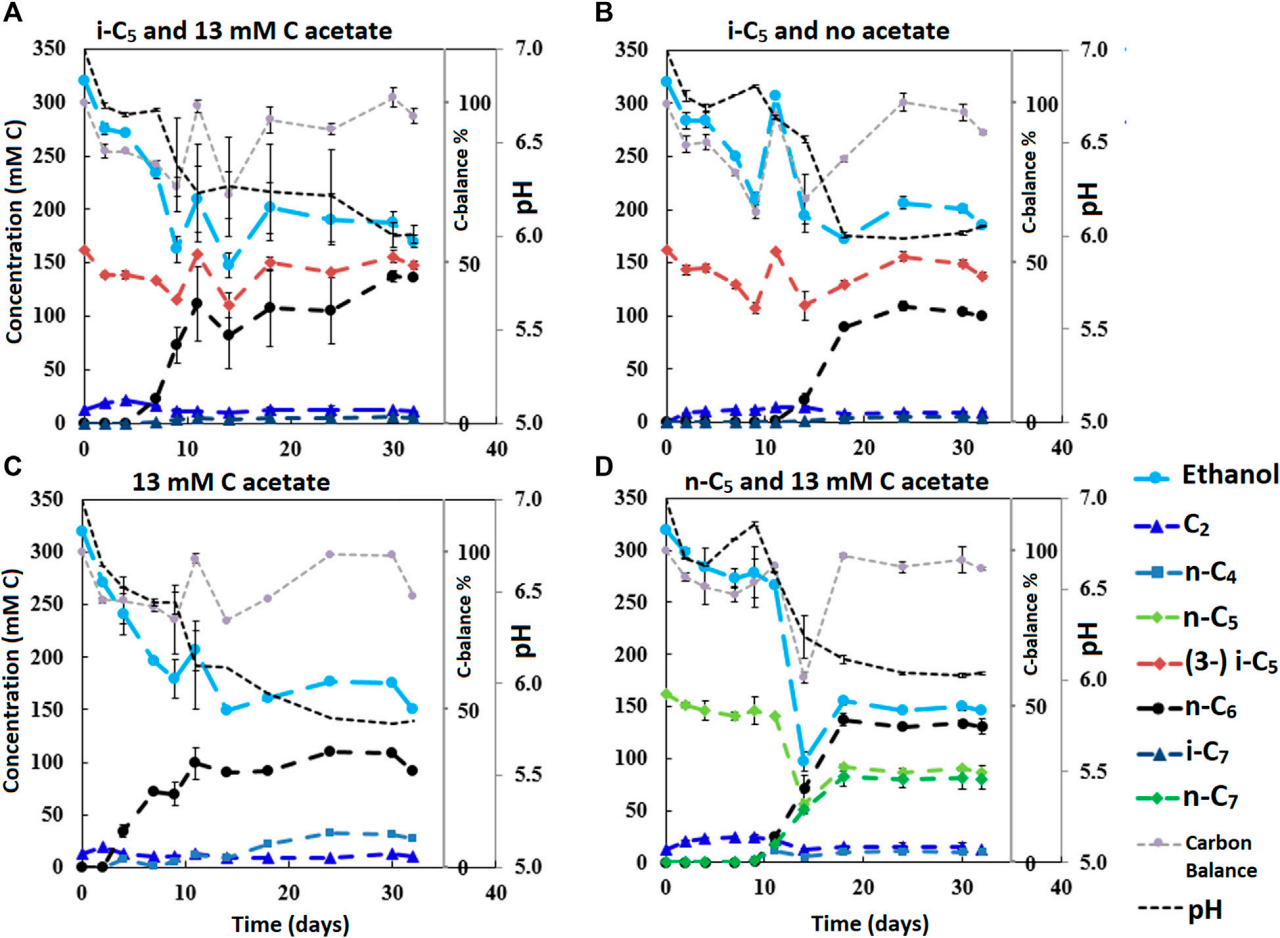
The four batches illustrate the effect of different initial acetate amounts and added carboxylates beside acetate on chain elongation activity. Complete absence of initial acetate **(B)** causes a prolonged lag phase, whereas the addition of a small amount **(A)** sped up the onset of chain elongation by approximately 7 days. Additionally, the batch without any additional carboxylate besides acetate shows an even faster onset of chain elongation activity **(C)**. The batch with added n-C_5_ **(D)** shows n-C_6_ and n-C_7_ formation and no i-C_7_ formation. Concentration profiles of components that were present in lower concentration ranges are shown in Supplementary Figure S9.

The dominant bioprocess throughout all batch series was straight chain elongation towards n-C_6_. Depending on the added carboxylate (i-C_4_, n-C_5,_ i-C_5_) besides acetate, varying amounts of alternative elongation products (i-C_6_, n-C_7_ and i-C_7_) were formed. The amount of acetate that was present in the beginning of the batch considerably affected the time it took for chain elongation to be observed. Concentration profiles of the second and third batch series are shown in Figures 2, 3, respectively. The carbon balances approach the value around 95% at the end of each batch, which is to be expected with these type of anaerobic fermentations (the remaining 5% is likely channeled into biomass formation).

It is relevant to emphasize that during all batches, except batch 2.B (where 65 mMC acetate was added), a large fraction of ethanol (58–71%) was not consumed and therefore still available as electron donor. Increasing the initial acetate concentration (2.B, Figure 2; Table 4) caused a substantial higher chain elongation activity towards n-C_6_ (2.6 times more n-C_6_ was formed compared to 2.A); more ethanol was consumed (only 5% remained) in this batch up to a higher hydrogen partial pressure (p_H2_) and higher final acetate concentration. However, only 3.1 mMC i-C_7_ was formed versus 6.4 mMC i-C_7_ in the control with a low initial acetate amount (2.A). A low acetate concentration in combination with a high hydrogen partial pressure (Roghair et al., 2018a; Spirito et al., 2018) hampered chain elongation activity in the first two series. In control batch 1.G the absence of BES allowed methanogenesis to consume CO_2_ down to a partial pressure < 1 kPa, concomitant with more excessive ethanol oxidation to acetic acid that led to a pH drop to < 5.7. These low CO_2_ partial pressure and pH values are limiting conditions for (ethanol-based) chain elongation bacteria (Barker and Taha, 1942; Tomlinson and Barker, 1954).

Alcohol formation was observed in all batches and showed a general trend that whenever a carboxylate is present, the corresponding alcohol is formed albeit up to a (10–50 times) lower concentration (i.e., i-C_4_ led to i-C_4_OH, n-C_6_ led to n-C_6_OH, etc.). The alcohol formation occurred in all batches up to a final concentration range between 1 and 5 mMC for each produced alcohols species. The concentration profiles of metabolites in this lower concentration range are shown for all batches in Supplementary Figures S4, S6, S7, S9. Gas headspace partial pressure profiles of all batches are shown in Supplementary Figures S5, S6, S8, S10. In general, the batches with added BES show accumulation of hydrogen gas during chain elongation activity until activity halts.

#### Chain Elongation of i-C_5_ Towards i-C_7_

The first batch series showed i-C_7_ formation in the batches fed with i-C_5_, albeit in very low amounts (Supplementary Figures S3, S4). The L/D 2-methylbutanoate racemate (L/D 2-MB) was seemingly not utilized at all as substrate for chain elongation, as no hypothetical elongation product (4-methylhexanoate, 4-MHA) was observed. A general trend was observed that the lower concentration batches showed a faster onset of chain elongation activity, compared to the higher concentration batches. Small amounts of branched pentanol formation were observed in all batches regardless which form of branched pentanoate was available. Due to a relatively high standard error of the branched C_5_ analysis compared to the low i-C_7_ and b-pentanol concentrations, it could not be determined whether i-C5 was molar-equally consumed. However, in all batches that did not contain i-C_5_ no i-C_7_ formation occurred. Additionally, the positive control batch without added yeast extract also showed i-C_7_ formation, excluding yeast extract conversion, as potential cause for i-C_7_ formation. The likely chain elongation of i-C_5_ towards i-C_7_ accounted to 7.2% (based on carbon atoms) of the formed compounds in the best performing batch regarding i-C_7_ formation (2.A). However, in this batch 98% of the supplied i-C_5_ still remained unconverted. The n-C_6_ accounted to 79.6% (based on carbon atoms) of formed compounds. Hexanol and iso-pentanol constituted to 4.7 and 0.6%, respectively, of the formed compounds. The final product selectivities (for i-C_7_, n-C_6_ and hexanol), and the percentages of unconverted ethanol for all other batches are shown in Tables 2–4.

## Discussion

### Acetate Availability and Branched Carboxylate Selectivity

The continuous reactor experiment was operated without any acetate in the influent with the intention to maximize i-C_4_ utilization during chain elongation and to maximize selectivity towards i-C_6_ formation. Compared to a previous study on i-C_6_ formation (de Leeuw et al., 2019), the current system achieved a 30% higher volumetric i-C_6_ (57 ± 4 mMC/day, or 1.1 ± 0.07 g/L/day) formation rate and a 70% higher average i-C_6_ broth concentration (125 ± 6.6 mMC, or 2.43 ± 0.13 g/L) in phase III. During the whole operation period the reactor was operating under apparent CO_2_ limited conditions (<1 kPa), meaning that the low availability limits chain elongation activity of well-known chain elongators such as *C. kluyveri* (Tomlinson and Barker, 1954). When the CO_2_ load in phase III was increased, overall chain elongation activity increased (n-C_6_ formation increase more than i-C_6_ formation). There was a higher (branched) i-C_6_ productivity, although selectivity towards i-C_6_ had dropped (from 27% in phase II to 20% in phase III). Higher *in situ* acetate formation, both directly via the chain elongation metabolism and via increased excessive ethanol oxidation led to increased straight chain elongation (see Supplementary Table S5). Homoacetogenesis cannot be fully excluded, however the low CO_2_ availability limits homoacetogenic activity that requires CO_2_ as electron acceptor.

The reactor behavior shows there is a tradeoff to be made when designing the system: 1) selectivity towards i-C_4_ elongation is high during acetate and CO_2_ limitation (which reduces overall chain elongation activity), or 2) straight chain elongation is stimulated by lifting the CO_2_ limitation leading to a decreased selectivity towards alternative carboxylate elongation. In all phases i-C_4_ was abundantly available, while acetate was only available via *in situ* formation. The sensitivity to increases in acetate show that there is a preference towards acetate as electron acceptor over i-C_4_ (and i-C_5,_ in the batches) within the established chain elongation microbiome. In the third batch series it was observed that CO_2_ was consumed down to low partial pressures (<1 kPa), while more H_2_ was consumed, and overall a higher percentage of ethanol was utilized for chain elongation compared to the previous batches; it suggests the microbiome had developed increased homoacetogenic activity.

### Low Affinity for i-C_5_ Elongation Suggests Co-Metabolism During Straight Chain Elongation

The degree by which i-C_5_ and i-C_4_ are elongated in a batch system varied. Formation of i-C_7_ contributed only 4.2% (based on carbon atoms) to the total produced compounds in the batch with both i-C_5_ and i-C_4_ (2.D). In contrast, i-C_6_ formation contributed for 27% to the total product spectrum, even though the molar concentration of i-C_5_ was higher than i-C_4_. With the L/D 2-methylbutanoate racemate batches no elongation product was observed at all and overall, the chain elongation rate diminished. Moreover, in the batch experiments a higher acetate availability negatively influences the selectivity towards branched chains, like what was observed in the continuous reactor. This is emphasized by the batches performed at 65 mMC and 6.5 mMC initial acetate. A higher initial acetate concentration (batch 2.B) increased total chain elongation activity, but considerably lowered the selectivity towards i-C_7_ (1.4%) compared to the control (7.3%) at low initial acetate amounts (batch 2.A). The results suggest the microbiome contains enzymes that can perform branched carboxylate elongation, but only to a certain degree. The varying selectivities can arise from two different scenarios: 1) acetate limitation and 2) no limitation (illustrated in Supplementary Figure S12). Hypothetically, the higher i-C_7_ selectivity during acetate limitation (2.A) can arise from kinetic impairment of acetate elongation at low acetate concentrations, while branched carboxylate elongation occurs of maximum speed. In contrast, at higher acetate concentrations (2.B) the alleviated kinetic impairment leads to more acetate elongation relative to branched chain elongation, causing a lower i-C_7_ selectivity.

The initially available acetate (6.5 mMC in 2.A versus 65 mMC in 2.B and 13 mMC in 3.A versus 0 mMC in 3.B) in the batch series greatly affected the time it took for chain elongation to occur. These results are in line with earlier studies that show a reduced chain elongation activity during acetate limitation (Spirito et al., 2018). Interestingly, all batches, except for 2.A where 65 mMC acetate was added, showed that first acetate formation started, before any chain elongation commenced (Supplementary Figures S4, S6, S7, S9). Despite presence of sufficient alternative electron acceptors, a minimum amount of acetate seems to be required for chain elongation to occur. The requirement of acetate hints towards a cometabolism for the branched electron acceptors within chain elongation, i.e., branched carboxylates are only elongated during straight chain elongation.

### Alcohol Formation as Electron Sink and Alternative Source for *in situ* Acetate Production

**FIGURE 4:**
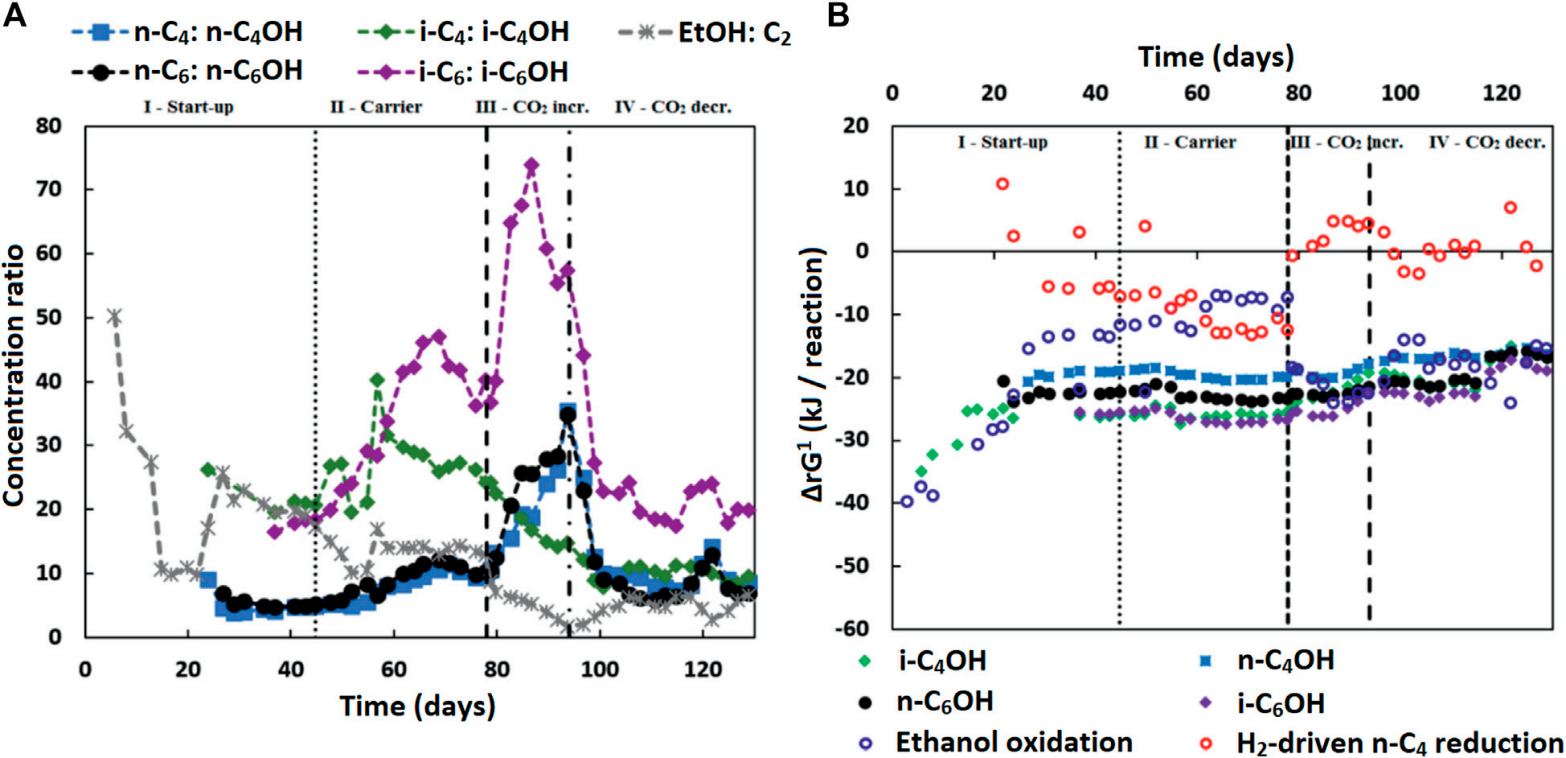
**(A)** The concentration ratios of carboxylates to corresponding alcohols (and ethanol to acetate) are shown. These ratios seem to be inversely correlated to the ethanol to acetate ratio, especially in the later phases (III and IV), except for the i-C_4_ to i-C_4_OH ratio. Isobutyrate was continuously fed into the reactor and present at high concentrations in the broth. This would contribute to the driving force of isobutanol formation, while lessening the inverse correlation of the i-C_4_ to i-C_4_OH ratio to the ethanol to acetate ratio. **(B)** The Gibbs free energy change (ΔrG^1^) is shown for the reactions: Ethanol+Carboxylate→Acetate+Corresponding Alcohol (for iC_4_OH, n-C_4_OH, n-C_6_OH and i-C_6_OH), direct ethanol oxidation and hydrogenotrophic butyrate reduction. The Gibbs free energy change was adjusted for the conditions (including pH and hydrogen partial pressure for ethanol oxidation and hydrogenotrophic reduction) in the reactor. The combined hydroxyl-carboxyl exchange reaction seems to remain stable in the range of -15–25 kJ reaction per reaction. The used Gibbs formation energies and assumption for the calculations are given in Supplementary Table S6.

The measured alcohol concentrations during the continuous reactor experiment followed a dependency on the concentrations of ethanol and acetate as well as on the concentration of the corresponding carboxylates (Figure 4A). This finding was in line with the earlier study where i-C_6_ and alcohol (i-C_4_OH, n-C_6_OH and i-C_6_OH) formation were found (de Leeuw et al., 2019). It suggests that excessive (hydrogenogenic) ethanol oxidation is coupled to (hydrogenotrophic) carboxylate reduction within the microbiome as shown in Table 6, resulting in a net carboxyl-hydroxyl exchange reaction. A coupling of reactions would imply that the thermodynamics driving force is no longer affected by pH and hydrogen partial pressure (p_H2_), in contrast to hydrogenotrophic carboxylate reduction to alcohols that is favored at a lowered pH and an elevated p_H2_ (See Supplementary Figure S2 for the p_H2_ in the continuous reactor) (Steinbusch et al., 2008). Figure 4B shows that after startup the ΔrG^1^ (corrected for measured concentrations) of the combined reactions for each carboxylate—alcohol pair (when correcting for the broth concentrations of the reactants and products) remained between 15–25 kJ per reaction. This value is close to the currently known minimum required energy gain for a catabolic reaction to sustain microbial growth (Jackson and McInerney, 2002), and suggests that this bioconversion could be utilized as energy-providing route by organisms growing in a specific niche. It still needs to be revealed which organism(s) play(s) a role in this alcohol formation.

**TABLE 6:** Thermodynamic calculations for ethanol oxidation and carboxylate (n-butyrate as example) reduction towards the corresponding alcohol (n-butanol). Δ_r_G^1^ indicates the reaction Gibbs free energy change at standard biological conditions (298°C, pH 7). Δ_r_G^2^ and Δ_r_G^3^ are corrected for reactants to products ratios (carboxylates and alcohols only) of 100 and 0.01, respectively.

Bioprocesses	Reaction	Δ_r_G^1^	Δ_r_G^2^	Δ_r_G^3^
Hydrogenogenic ethanol oxidation	$CH_{3}CH_{2}OH+H_{2}O\to CH_{3}COO^{-}+2H_{2}+H^{+}$	*8.35*	−3.07	19.76
Hydrogenotrophic carboxylate reduction (butyrate)	$C_{3}H_{7}COO^{-}+2H_{2}+H^{+}\to C_{3}H_{7}CH_{2}OH+H_{2}O$	−16.15	−27.56	−4.73
Combined: Hydroxyl-carboxyl exchange	$C_{3}H_{7}COO^{-}+CH_{3}CH_{2}OH\to C_{3}H_{7}CH_{2}OH+CH_{3}COO^{-}$	−7.80	−30.63	15.03

Possibly chain elongation microorganisms themselves are solely responsible for the formation of the longer alcohols. It is reported that *Clostridium kluyveri*, a well-known chain elongator, is able to produce small amounts of higher alcohols (Kenealy and Waselefsky, 1985). A batch series performed using pure *Clostridium kluyveri* with propionate and ethanol under different hydrogen pressures showed that propanol formation increased with an increasing p_H2_ (Candry et al., 2020). The alcohol formation during acetate limitation in combination with a high p_H2_ could hypothetically be method to get rid of excess electrons when chain elongation-coupled ethanol oxidation is thwarted due to high hydrogen partial pressures. Carboxylate reduction then replaces hydrogen formation as electron sink.

Alternatively, another specialized organism performing the hydroxyl-carboxyl exchange could be present. It would require an organism similar to *Clostridium autoethanogenum* (Diender et al., 2016) that can harvest the energy from ethanol-derived electrons via an energy-coupled transhydrogenase (Rnf complex) (Westphal et al., 2018) before reducing the carboxylates to alcohols. A second alternative would be syntrophic interaction between ethanol oxidizers and “hydrogenotrophic” carboxylate reducers [via H_2_ exchange and/or Direct Interspecies Electron Transfer (DIET) (Nozhevnikova et al., 2020)]. Although the thermodynamic calculations performed with macroscopic data show that the hydrogenotrophic carboxylate reduction is often unfeasible (Figure 4B), a syntrophic coupling of ethanol oxidation and carboxylate reduction would imply that the actual microscopic conditions are such that both (in syntophy-growing) microorganisms are able to proliferate (Smith and McCarty, 1989).

The gradual increase of i-C_4_OH formation during phase III and the increased alcohol formation in phase IV compared to phase II indicate that this additional bioconversion capability had slowly become more prominent within microbiome. Consequently, the p_H2_ did not recover in phase IV to the earlier values in phase II (9.2 ± 1.3 kPa) after reducing the CO_2_ load; it was kept in a lower range (0.6 ± 0.4 kPa in phase IV) by the microbiome, while alcohol formation spiked. The alcohol formation likely acted as an alternative electron sink when methane formation had dropped due to the sudden lower availability of CO_2_, as was also observed previously (de Leeuw et al., 2019).

The onset of the alcohol formation implies that the earlier achieved high selectivity towards i-C_6_ in phase II could be transient. A low acetate concentration is used as steering parameter in this research to increase the selectivity towards i-C_4_ elongation. However, in combination with high ethanol and high other carboxylate amounts, a low acetate concentration leads to a thermodynamic potential that allows an alternative source of *in situ* acetate formation via hydroxyl-carboxyl exchange.

### Outlook for Further Bioprocess Development

Chain elongation microbiomes can be engineered to produce various chemicals depending on the supplied feedstock and steered reactor conditions. The higher branched and straight alcohol formations described in this research seem to be thermodynamically dependent on the reactant to product ratio. If the products could be removed *in situ* this could drive the reaction towards more straight and branched alcohol formation. Increasing the alcohol formation in this way could lead to an interesting biochemical production process in itself; the observed alcohol titers are in a suitable range for *in situ* extraction via gas stripping (Richter et al., 2016). This method may be used to develop processes that upgrade the ethanol in dilute ethanol-containing residue streams to higher alcohols.

Branched carboxylates such as i-C_4_ and apparently also i-C_5_ can be used as electron acceptor during chain elongation fermentations with a varying extend of conversion. Operating the reactor under acetate and CO_2_ limited conditions increases the selectivity towards branched carboxylate elongation, but as a tradeoff overall chain elongation activity is reduced. The conversions of branched carboxylates to longer chains seem only to occur as a form of co-metabolism during straight chain elongation. It remains to be seen if the co-metabolism, that is expressed as a dependency on straight chain elongation activity, can be lifted. Acetate plays a pivotal role within the chain elongation metabolism as it can both serve as a primer and elongation (acetyl-CoA) unit (Schoberth and Gottschalk, 1969). However, research has already shown that it is possible to increase the affinity of butyrate relative to acetate for an engineered thiolase (Bonk et al., 2018), as well as first efforts to modify thiolases to use branched carboxylates as primers (Clomburg et al., 2018). Further efforts to tailor the thiolase and other involved enzymes via metabolic engineering could offer perspectives where the k_cat_ and K_m_ values for branched carboxylates and their conversion intermediates are increased. This metabolic engineering approach would require pure or coculture cultivation of the responsible bacteria, in contrast to the presented research. Aside from bioconversion kinetics, the metabolic fluxes could also have been hampered by uptake and export limitations.

Production of i-C_7_ in the observed amounts in this study at this stage are unattractive for direct industrial applications compared to the formation of n-C_6_. Still with fractional distillation of the produced broth considerable amounts of i-C_7_ may be obtained. In the batch experiments i-C_5_ is hardly elongated (∼98% remains unconverted) in the cases where it is supplied in excess and acetate is only present in low amounts. So far, it is remarkable that i-C_4_ elongation has different kinetics compared to i-C_5_ elongation. It shows that the involved microbiome has not developed fully optimized enzymes for the artificially imposed selective pressure with low amounts of acetate and large amounts of alternative electron acceptors. In addition to metabolic engineering approaches further research on selection pressure and natural adaptation in open culture microbiomes can provide a potential i-C_7_ bioprocess development utilizing organic residual streams.

## Data Availability

The raw data supporting the conclusion of this article will be made available by the authors in the DANS EASY research database with doi:10.17026/dans-x2v-xeta.
