# Supplementary material for: Open Culture Ethanol-Based Chain Elongation to Form Medium Chain Branched Carboxylates and Alcohols
Source: Front Bioeng Biotechnol. 2021 Aug 17;9:697439. doi: 10.3389/fbioe.2021.697439 (PMC8416115; doi:10.3389/fbioe.2021.697439)
Supplement: Supplementary file 1 [file DataSheet1.docx]

Supplementary Material

**Open culture ethanol-based chain elongation to form medium chain branched carboxylates and alcohols**

**Kasper D. de Leeuw, Theresa Ahrens, Cees J.N. Buisman, David P.B.T.B. Strik***

Environmental Technology, Wageningen University & Research, Bornse Weilanden 9, 6708 WG, Wageningen, The Netherlands

## Material and Methods

***Medium composition***

The basic feed recipe is shown in Table S1 and remained the same throughout the experiments. The composition of the used stock solutions with vitamin B and trace elements are listed in Table S2, Table S3 and Table S4.

**Table S1.** Basic feed recipe used for the reactors and with small alterations also for the batch experiments. Composition of the vitamin B and trace element solutions can be found in Table S2, Table S3 and Table S4. Stock solutions I and II were made resulting in the final listed concentrations in the medium.

| **Compound** | | **Concentration g/l** |
| --- | --- | --- |
| Sodium Acetate trihydrate | | *varying* |
| Yeast extract | | *varying* |
| NaOH pellets | | *1.1* |
| Ethanol (Abs) (liquid) | | *varying* |
| i-C4 (liquid) | | *varying* |
| Stock I (50x) | *NH_4_H_2_PO_4_* | *3.6* |
|  | *MgCl_2_*6H_2_O* | *0.33* |
|  | *MgSO_4_*7H_2_O* | *0.2* |
| Stock II (50x) | *CaCl_2_*2H_2_O* | *0.2* |
|  | *KCl* | *0.15* |
| Vitamin solution | | *1 ml* |
| Trace elements, I & II | | *0.5 ml* |

**Table S2.** Composition of the “Vitamin solution” 1000x concentrated.

| **Compound** | **Concentration (g/L)** |
| --- | --- |
| Biotin | *0.106* |
| Folic acid | *0.005* |
| Pyridoxal-HCl | *0.0025* |
| Lipoic acid | *0.015* |
| Riboflavin | *0.0125* |
| Thiamine HCl | *0.266* |
| Ca-D-Pantothenate | *0.413* |
| Cyanocobalamin (Vit. B12) | *0.0125* |
| P-aminobenzoic acid | *0.0125* |
| Nicotinic acid | *0.0125* |

**Table S3.** Composition of the “Trace elements I” solution 2000x concentrated

| **Compound** | **Concentration (g/L)** |
| --- | --- |
| FeCl_2_*4H_2_O | *30* |
| HCl (1 M) | *77* |
| MnCl_2_*4H_2_O | *0.6* |
| H_3_BO_3_ | *6.0* |
| CoCl_2_*6H_2_O | *4.0* |
| CuCl_2_*H_2_O | *0.2* |
| NiCl_2_*6H_2_O | *0.4* |
| ZnSO_4_*7H_2_O | *2.0* |

**Table S4.** Composition of the “Trace elements II” solution 2000x concentrated.

| **Compound** | **Concentration (g/L)** |
| --- | --- |
| Na_2_MoO_4_*2H_2_O | *0.6* |
| Na_2_SeO_3_ | *0.2* |
| NaOH (4 M) | *3.1* |

**Step by step protocol for the batches**

1. Add to a 500 ml beaker in this order (plan for minimal waste):

- 0.25 g yeast extract (except batch 1.H)
- Ethanol, i-C_5_, acetate, i-C_4_ were added according to Table 1 and Table 2 in the main manuscript: 50/25 mL 1.6M ethanol solution, 50/25 mL 65mM sodium acetate trihydrate, 100/50 mL 325 mM (2-) / (3-) i-C_5._
- 10 ml stock I (see composition in Table S1)
- 10 ml stock II (see composition in Table S1)
- 100 mL 50 g/L BES solution (except batch 1.G)
- 0.5 ml vitamin solution (see composition in Table S2)
- 0.25 ml trace element I solution (see composition in Table S3)
- 0.25 ml trace element II solution (see composition in Table S4)

1. Add demi water up to 400 ml.
2. Set pH to 6.5 (4M KOH)
3. Transfer to a 500 ml flask
4. Add 5 ml re-suspended cells.
5. Add demi water up to 500 ml.
6. Stir shortly.
7. Transfer 150 ml to each batch bottles
8. Take a sample from the batch reactors (5 ml)
9. Cap the batches.
10. Replace the headspace at 1.5 bar with a gas exchanger, composition was 90 % N_2_ and 10 % CO_2_ for the first batch series (Table 1) and according to Table 2 for the second batch series. Add one extra bottle in each cycle.
11. Measure the absolute pressure.
12. Carry out headspace gas composition analysis of the extra bottle.
13. Place the bottles in a 35°C shaking incubation cabinet

***Branched C_7_ carboxylate determination***

*
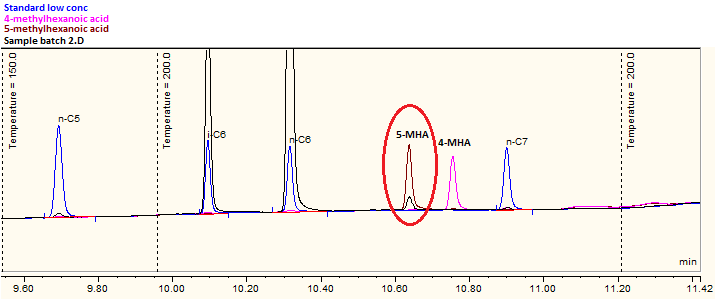
*

**Figure S1.** Chromatogram showing the branched heptanoate produced is 5-methylhexanoate (indicated in picture as 5-MHA). 4-MHA clearly has a different retention time and was not observed as product throughout the experiments.

**Carbon and Electron balances**

The carbon and electron balances were calculated via the following expressions:

Continuous experiment: $Carbon balance \left( \% \right)=\frac{Carbon out}{Carbon in}\cdot100\%$

With $Carbon in=\sum_{n} (Flow_{liquid, in}*C_{n,in})+\sum_{i} (Flow_{gas,in}*x_{i,in})$

and $Carbon out=\sum_{n} (Flow_{liquid, out}*C_{n,out})+\sum_{i} (Flow_{gas,out}*x_{i,out})$

Batch experiments $Carbon balance \left( \% \right)=\frac{{Carbon}_{t=x}}{{Carbon}_{t=0}}\cdot100\%$

With ${Carbon}_{t=0}=\sum_{n} (V_{t=0}*C_{n,t=0})+ \sum_{i} ({pressure}_{t=0}*x_{i,t=0})$

and ${Carbon}_{t=x}=\sum_{n} (V_{t=0}*C_{n,t=x})+ \sum_{i} ({pressure}_{t=x}*x_{i,t=x})$

With C_n,in_, C_n,out_, C_n,t=0_, C_n,t=x_ being the concentrations in mM C of components containing carbon and x_i,in_, x_i,out_, x_i,t=0_, x_i,t=x_ being the mole fractions for carbon containing compounds such as CO_2_ and CH_4_.

Note that the balances for the batches were calculated assuming constant volume.

The electron balances were calculated in a similar fashion, considering the degree of reduction of the components (for gases then H_2_ is then also included with y=2 electrons per mole), instead of the carbon content.

## Results and Discussion

*
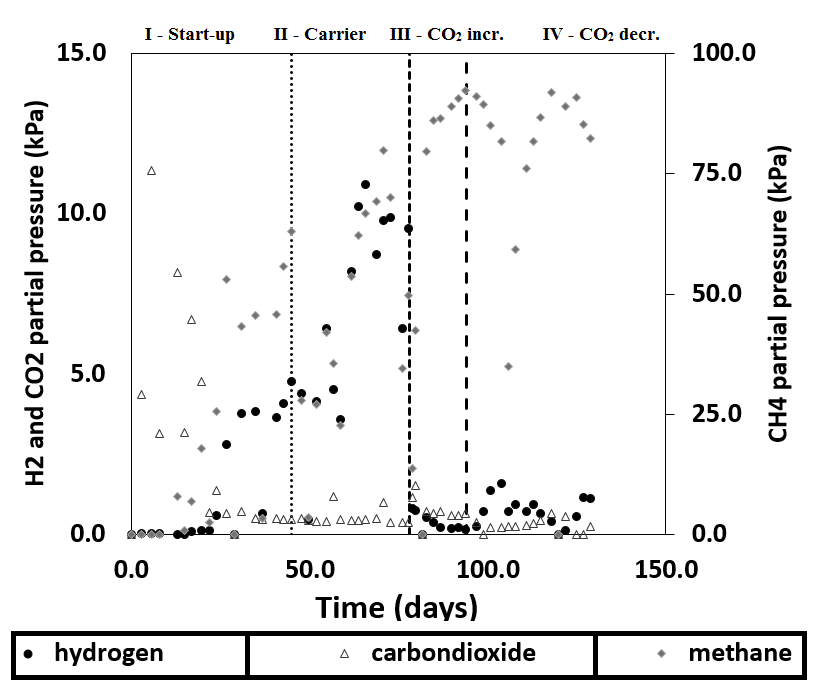
*

**Figure S2.** Gas partial pressures within the continuous reactor headspace.

*
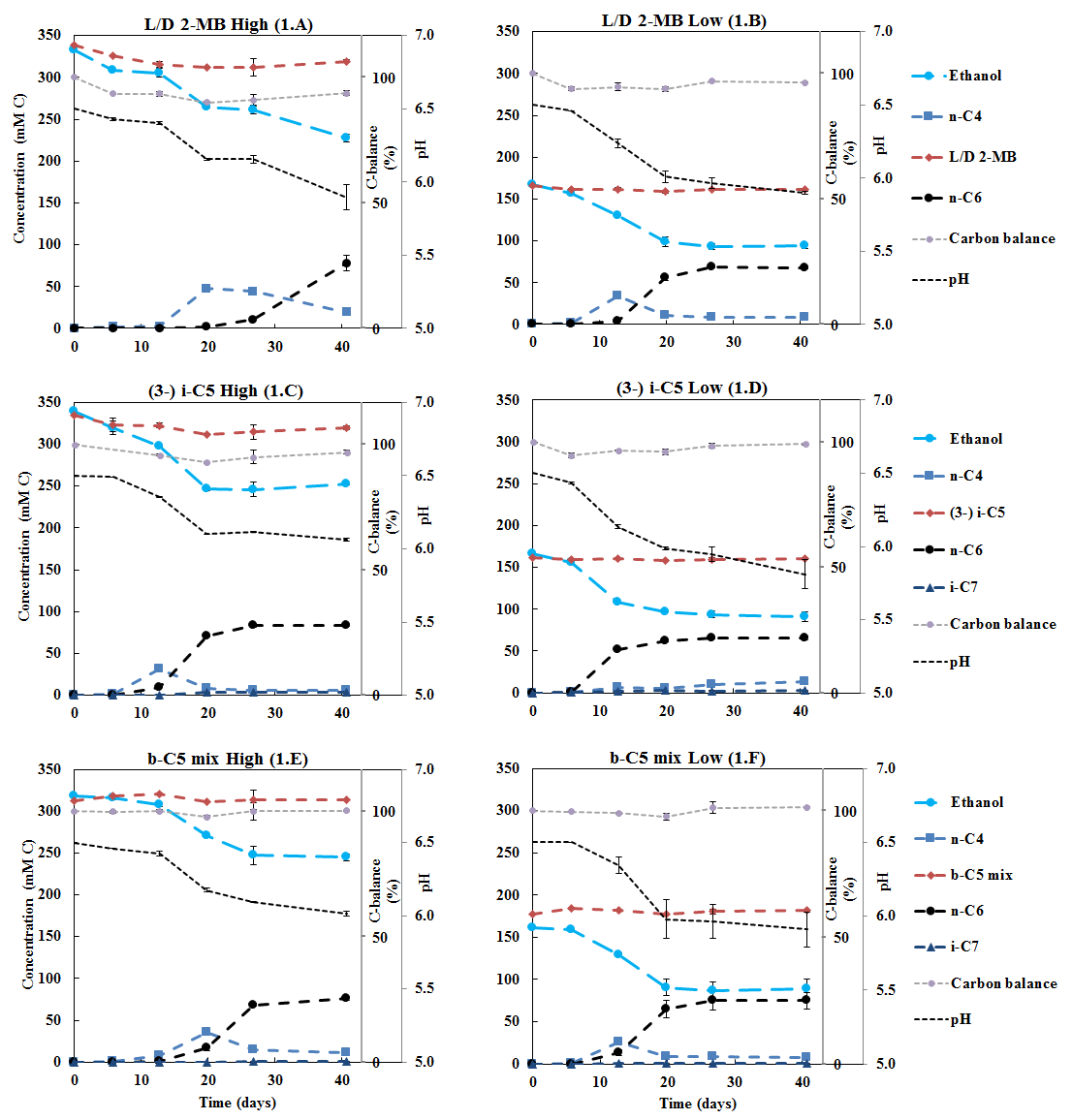
*

**Figure S3.** The graphs show the concentration profiles of batch 1.A to 1.F. Concentration profiles of the metabolites that were present in very low concentrations (e.g. acetate, n-hexanol, b-pentanol as well as i-C_7_ in a range between 0 and 25 mM C) are shown in Figure S4. Headspace compositions are shown in Figure S5. Concentration profiles of the control batches are shown in Figure S6.

*
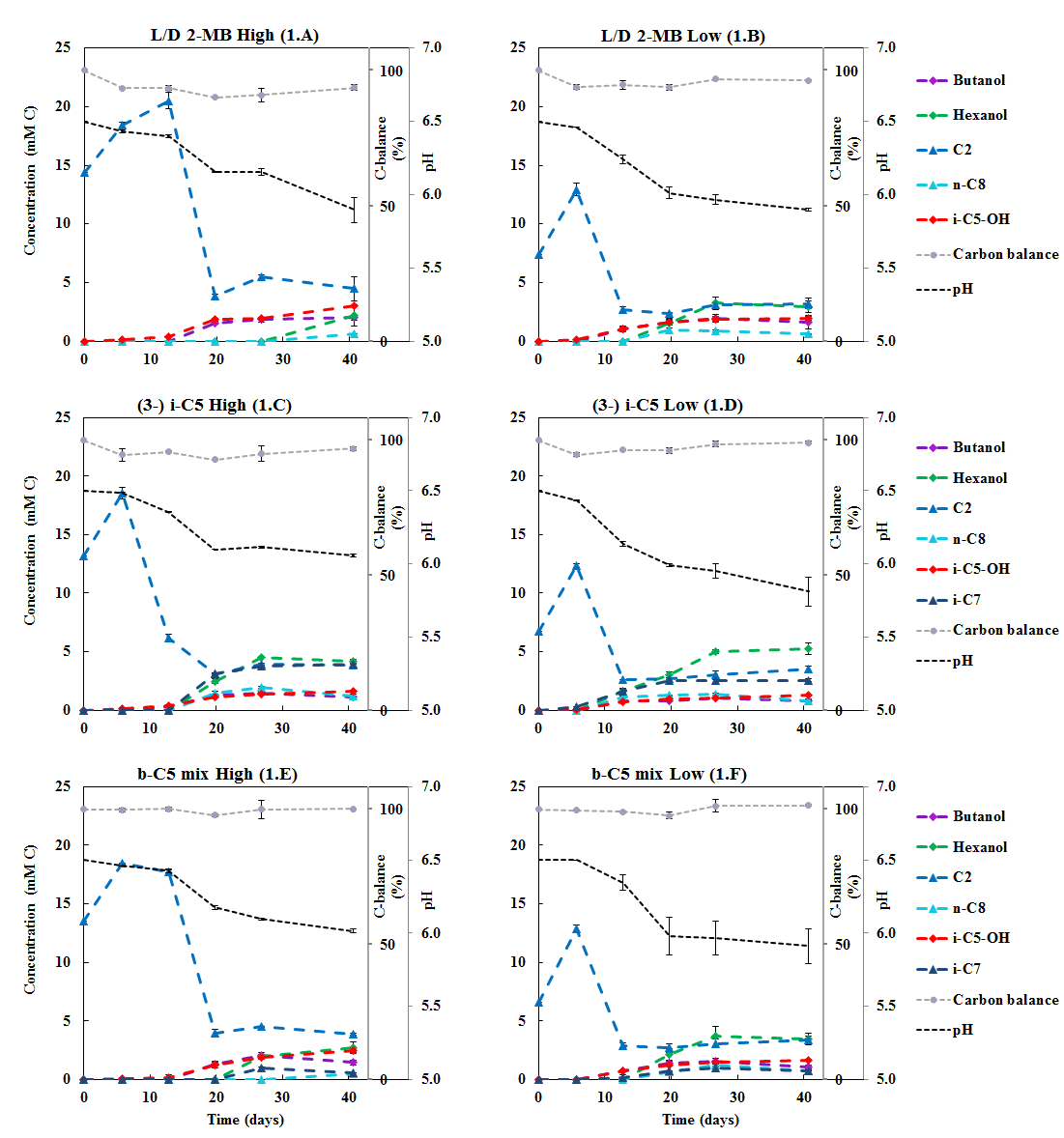
*

**Figure S4.** Concentration profiles of the first batch series for metabolites that were present at low concentrations.

*
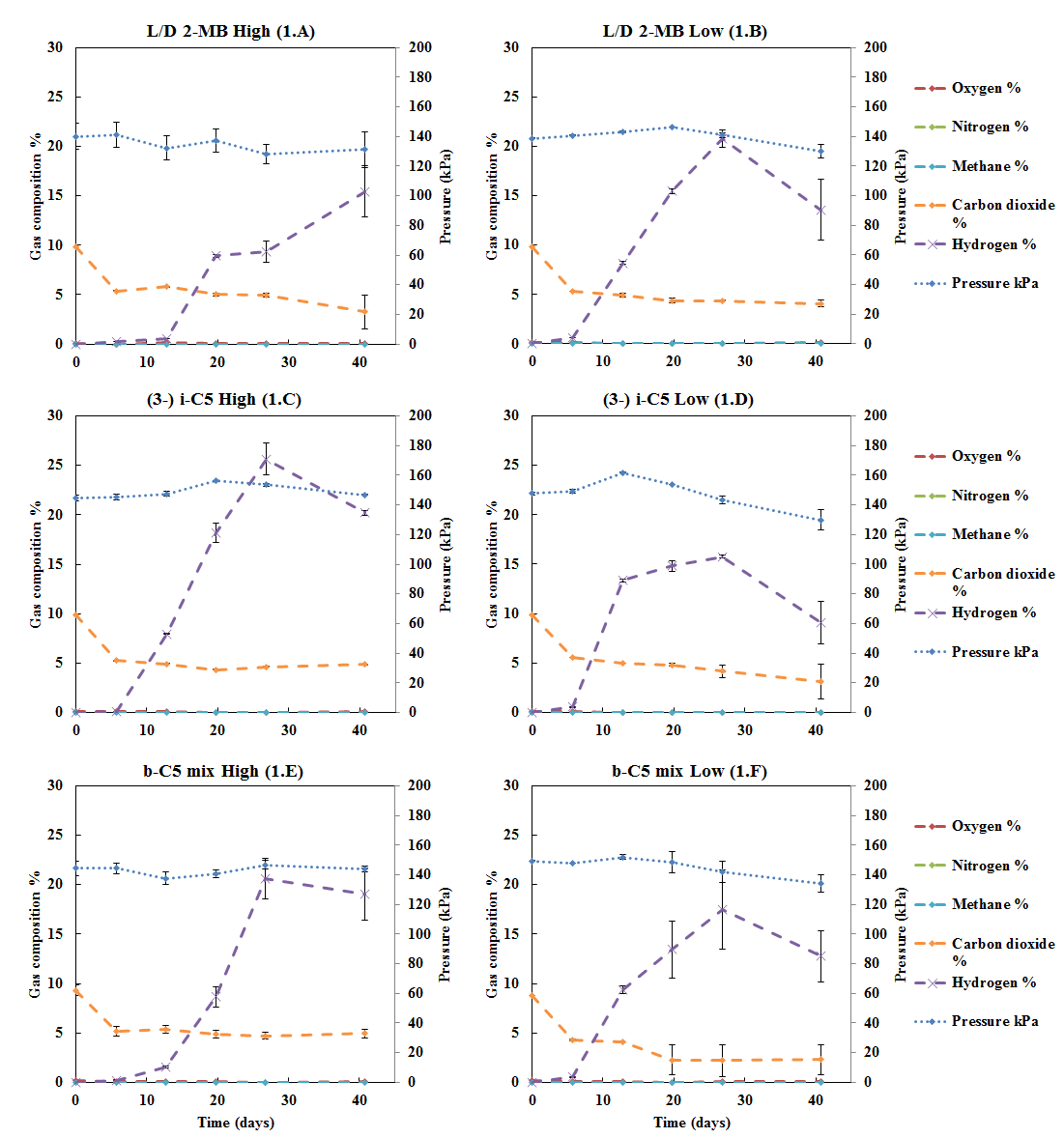
*

**Figure S5.** Headspace compositions and pressures of the first batch series. Both methane and oxygen overlap at around 0 %.

*
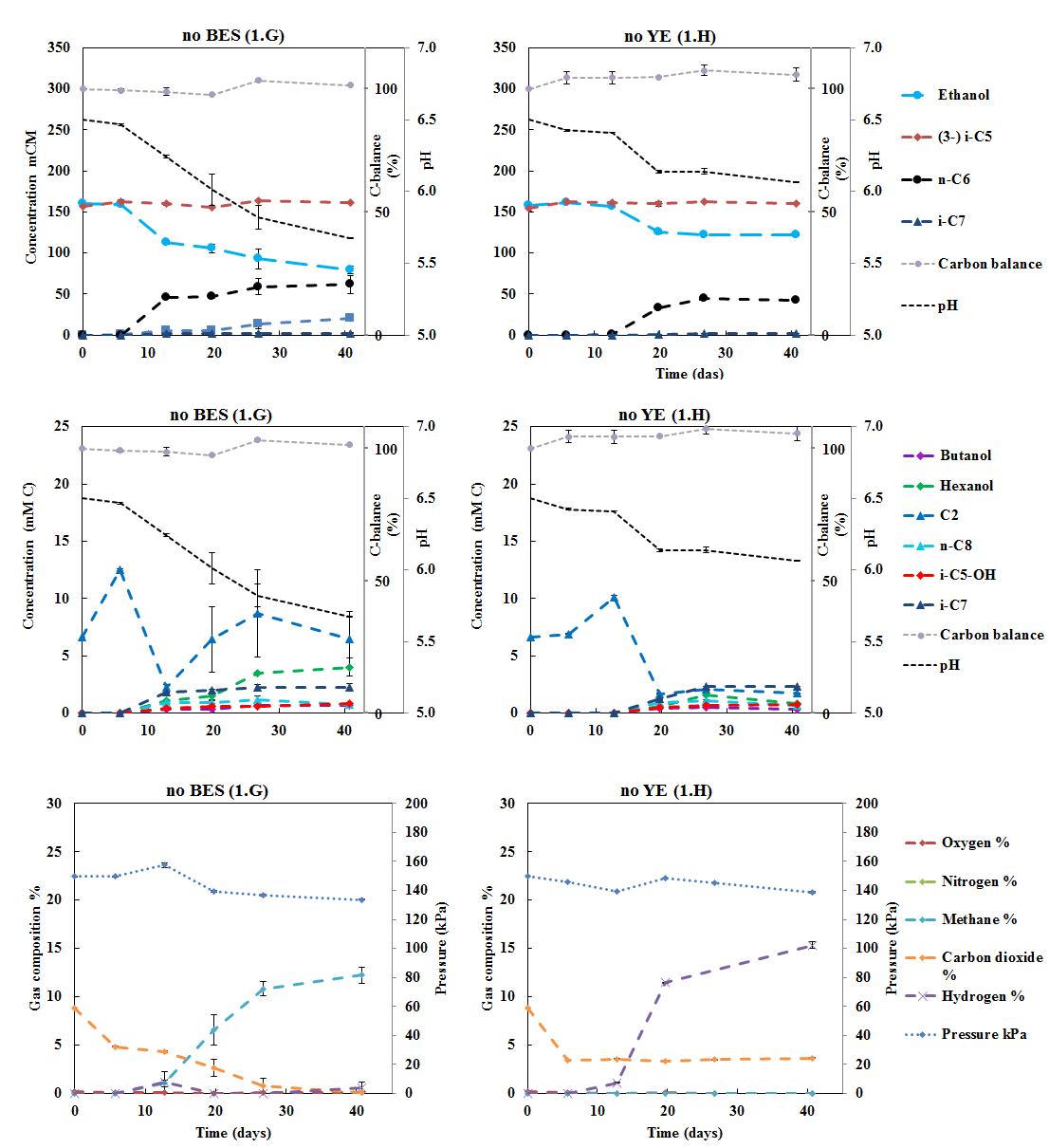
*

**Figure S6.** Concentration profiles and headspace compositions of the two negative controls where BES was left out (1.G) and where yeast extract was left out (1.H).


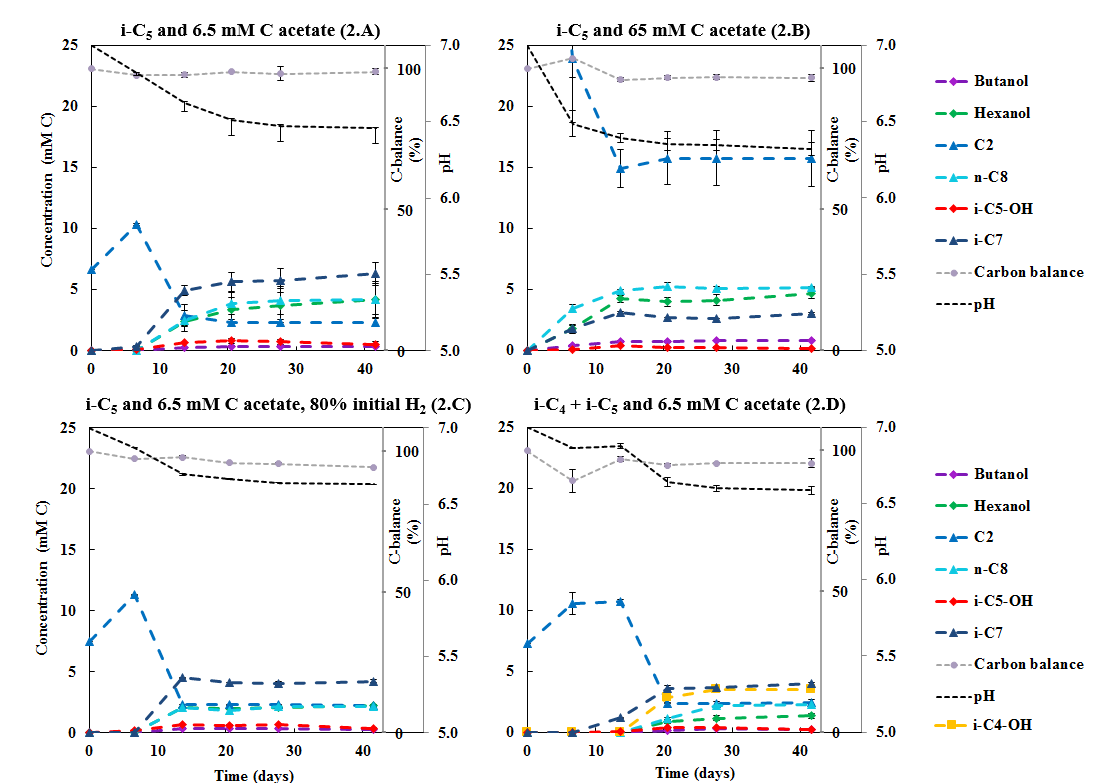


**Figure S7.** Concentration profiles of the second batch series for metabolites that were present at low concentrations. Initial acetate concentration for batch 2.B was 65 mM C and cannot be shown with this axis configuration. In Figure 2 of the main manuscript the full progression of acetate is shown.


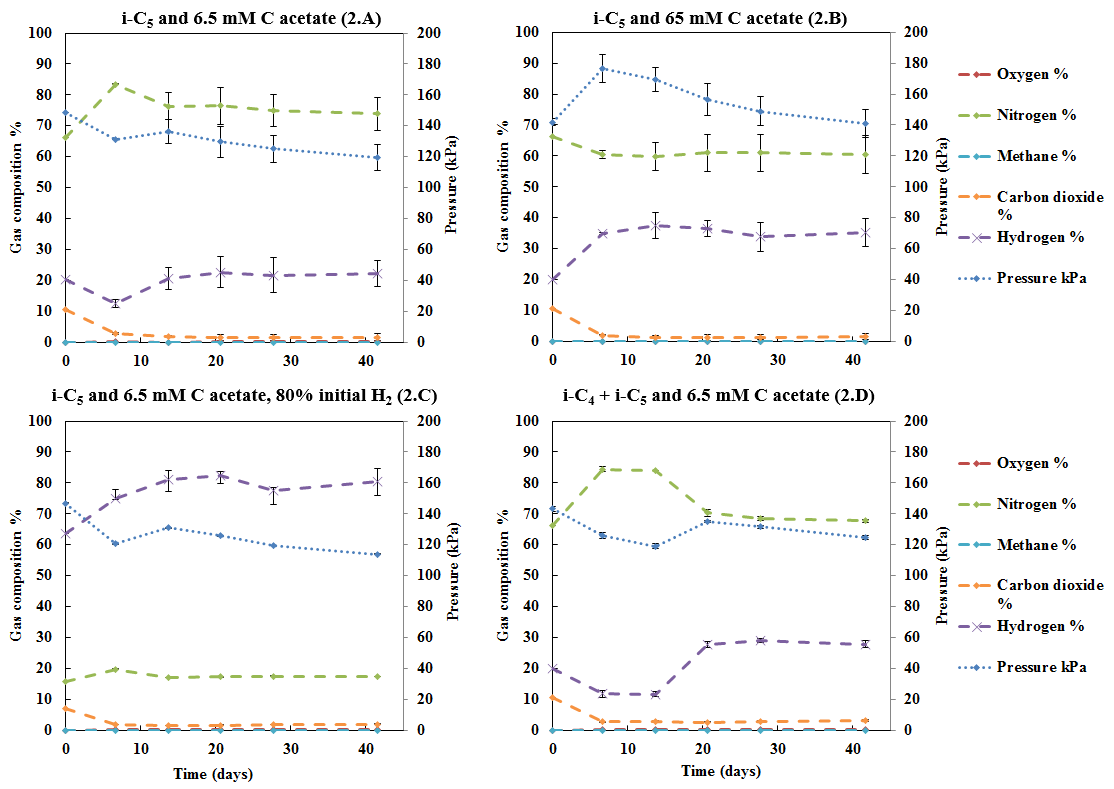


**Figure S8.** Headspace compositions and pressures of the second batch series. Both methane and oxygen overlap at around 0 %.

*
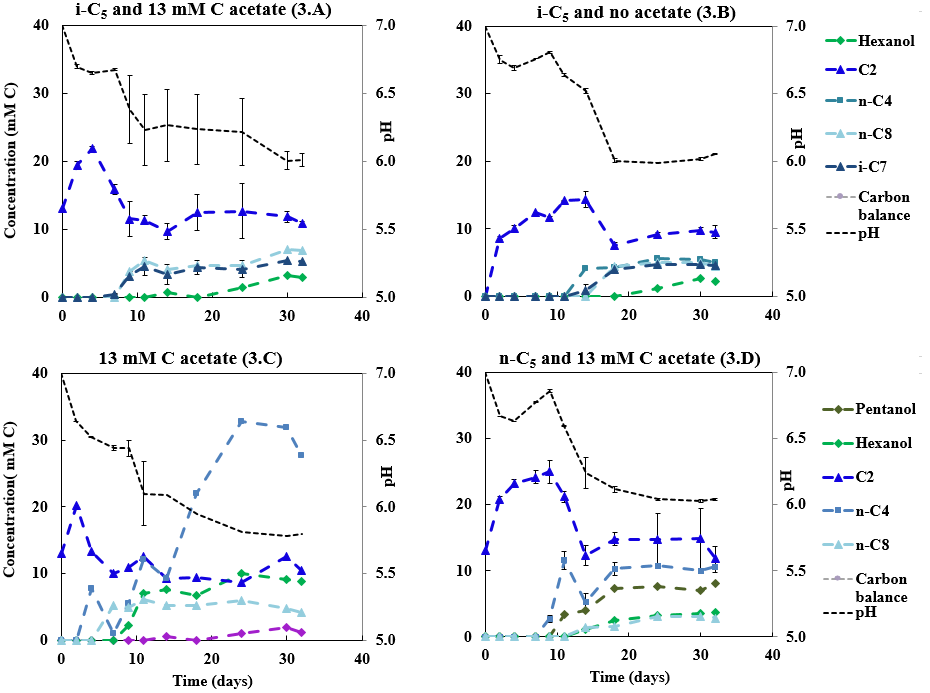
*

**Figure S9.** Concentration profiles of the third batch series for metabolites that were present at low concentrations.

*
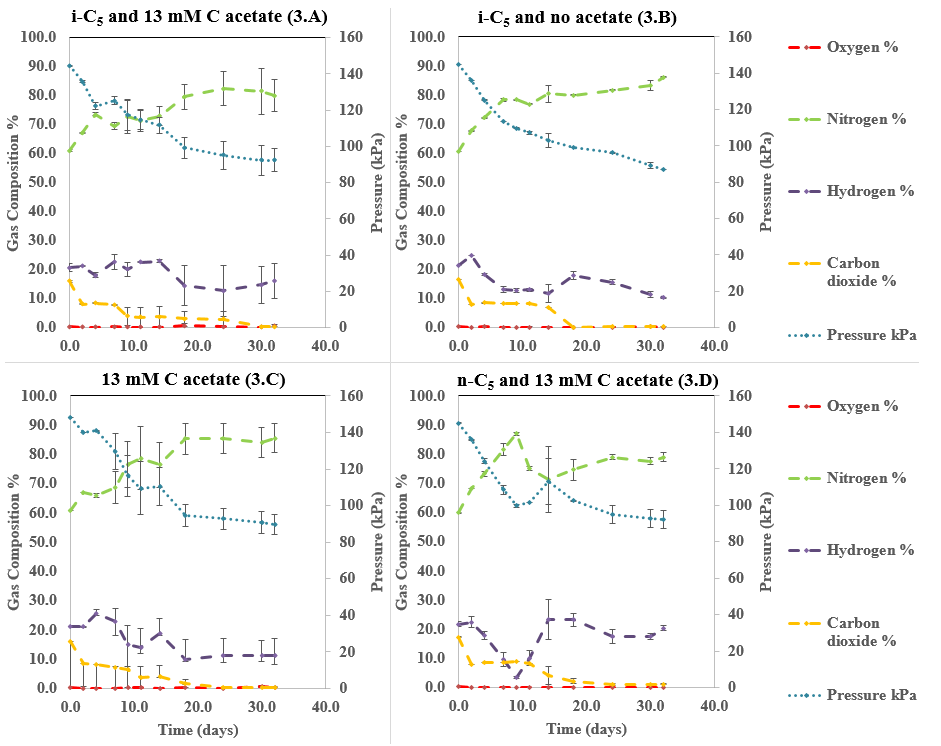
*

**Figure S10.** Headspace compositions and pressures of the third batch series.

**Table S5.** Stoichiometric analysis of ethanol consumption and calculated in situ acetate formation compared to measured straight chain formation. The average values given in Table 5 were used for the calculations. All calculated in situ formed acetate should stoichiometrically match with the measured amount of straight carboxylate and straight longer alcohol chain (Measured straight chain elongation / Calculated acetate formation should approach 100%).

|  | Consumed Ethanol (mmol L-1 day-1) | | | Calculated acetate formation (mmol L-1 day-1) | | | Measured straight chain formation, e.g. C2, nC4, nC6, nC8, nC4OH, nC6OH (mmol L-1 day-1) | Measured straight chain elongation / Calculated acetate formation |
| --- | --- | --- | --- | --- | --- | --- | --- | --- |
| Period | Total EtOH consumption | via CE (5x, 1x) | Via EEO (% of total EtOH consumption) | from CE/6 | from EEO | Total |  |  |
| Phase II | 67 | 53 | 14 | 8.8 | 14 | 23 | 27 | 116% |
| Phase III | 110 | 82 | 27 | 14 | 27 | 41 | 39 | 96% |
| Phase IV | 102 | 57 | 45 | 9.5 | 45 | 54 | 34 | 62% |
| Used stoichiometries | | | | | | | | |
| Combined Ethanol based chain elongation (example for acetate)  Reverse beta-oxidation (5x)  Coupled ethanol oxidation (1x) | | | | **$6CH_{3}CH_{2}OH+4CH_{3}COO^{-}\to5C_{3}H_{7}COO^{-}+2H_{2}+4H_{2}O+H^{+}$** $5CH_{3}CH_{2}OH+5CH_{3}COO^{-}\to5C_{3}H_{7}COO^{-}+5H_{2}O$ $CH_{3}CH_{2}OH+H_{2}O\to CH_{3}COO^{-}+2H_{2}+H^{+}$    | | | | |
| (Excessive) Direct ethanol oxidation | | | | $CH_{3}CH_{2}OH+H_{2}O\to CH_{3}COO^{-}+2H_{2}+H^{+}$ | | | | |

*Note: Ethanol consumption via CE or via EEO, as well as consequent acetate formation was calculated with formulas presented by Roghair et al. (2018) and de Leeuw et al. (2019) (1, 2). For the calculations the following stoichiometry for chain elongation was assumed: 5 ethanol is used for the reverse beta oxidation pathway, whereas 1 ethanol is used for oxidation towards acetate and hydrogen gas(3). Several uncertainties are present in the analysis due to (i) unaccounted yeast extract consumption, (ii) unaccounted acetate formation via e.g. homoacetogenesis, (iii) an accumulative effect of standard deviation multiplication (not shown) and (iv) the inaccurate C-balance for phase IV (87 ±10%). EEO calculations using concentrations when the C-balance is lower than 90% (due to measurement errors) can cause a large error which propagates through the whole stoichiometric analysis (see Figure S11).*

*
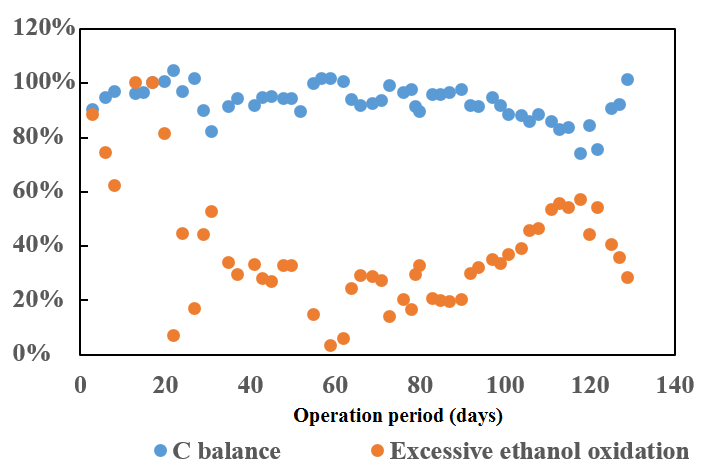
*

**Figure S11.** Carbon balance of the continuous reactor and calculated excessive ethanol oxidation. When the carbon balance drops below ~90% around day 100, the calculated excessive ethanol oxidation rises increasingly. The explanation for this overestimated EEO is a mismatch between the measured ethanol and fatty acids compared to what would be expected from the stoichiometry that is used to calculate the EEO. It indicates that concentration measurements (for ethanol in particular) might have been too low for phase IV, leading to an overestimation of EEO and a resulting mismatch in observed CE / calculated C2 formation.

*
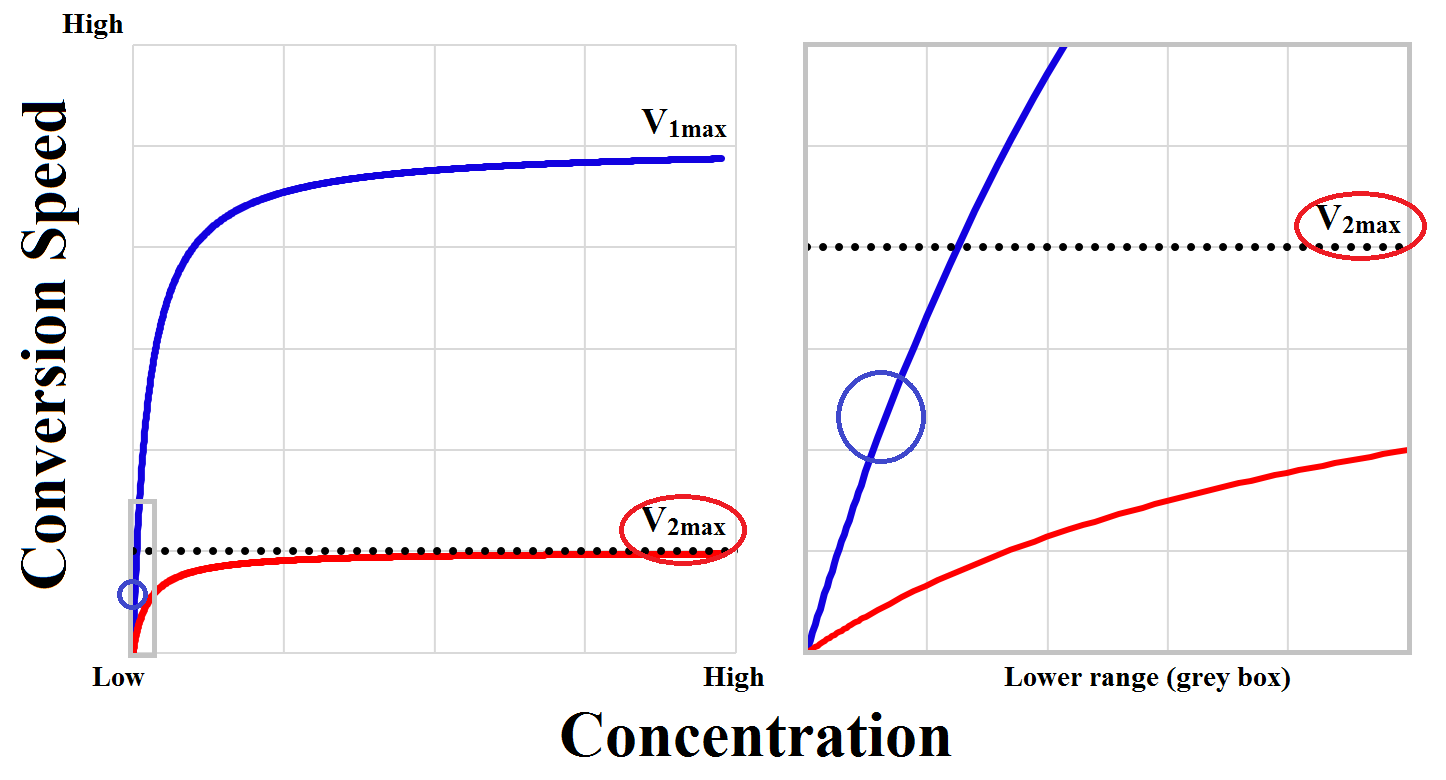
*

**Figure S12.** This figure illustrates a simplified interpretation (non-cooperative, Michaelis-Menten) of the possible enzyme kinetics at play when acetate is present at a low concentration (V_1_, blue), while a branched carboxylate (V_2_, red) is present at a high concentration. Values in this graph are arbitrarily chosen and therefore not shown; the only assumption made is that V_1_max > V_2_max. The circles show where the conversion speeds would be under acetate limited conditions, V_1_ being severely limited while V_2_ would be operating at maximum rates. At high acetate concentrations the relative speeds would be different leading to a lower branched carboxylate elongation selectivity.

**Table S6.** Overview of Gibbs formation energies for components used to calculate the Gibbs reaction energies of the hydroxyl-carboxyl exchange reactions (4). The Gibbs formation energies are based on chemical group contributions (5). The formation energies of n-hexanol, isohexanoate and isohexanol could not be found. However, during the hydroxyl-carboxyl exchange reactions, the contribution to the reaction Gibbs energy difference comes from the difference between the ΔGf^0^ of acetate/ethanol and the ΔGf^0^ of the larger carboxylate/alcohol pairs. The lengths of the hydrated carbon atoms tails for n-pentanoate/n-pentanol and n-octanoate/n-octanol hardly contribute to the ΔGf^0^ (-177.6 and 176.6 kJ mol^-1^ respectively) and the resulting Δ_r_G^0^ (-11.3 and -12.3 kJ mol^-1^ respectively). Therefore, the Δ_r_G^0^ of the n-hexanoate to n-hexanol and the isohexanoate to isohexanol hydroxyl-carboxyl exchange reactions was assumed to be -11.3 kJ mol^-1^, in line with the hydroxyl-carboxylate exchange reactions for n-pentanoate and n-octanoate.

| *Component* | *Gf^0^* | *ΔGf^0^* | *Reaction* | *Δ_r_G^0^* |
| --- | --- | --- | --- | --- |
|  | *kJ mol^-1^* | *kJ mol^-1^* |  | *kJ mol^-1^* |
| *Acetate* | *-367.9* |  |  |  |
| *Ethanol* | *-179.0* | *-188.9* |  |  |
| *butyrate* | *-352.6* |  | *hydroxyl-carboxylate exchange n-C_4_ + EtOH 🡪 n-C_4_OH + C_2_* | *-7.8* |
| *n-butanol* | *-171.5* | *-181.1* |  |  |
| *i-butyrate* | *-340.9* |  | *hydroxyl-carboxylate exchange i-C_4_ + EtOH 🡪 i-C_4_OH + C_2_* | *-11.3* |
| *i-butanol* | *-163.3* | *-177.6* |  |  |
| *n-pentanoate* | *-336.6* |  | *hydroxyl-carboxylate exchange n-C_5_ + EtOH 🡪 n-C_5_OH + C_2_* | *-11.3* |
| *n-pentanol* | *-159.0* | *-177.6* |  |  |
| *n-octanoate* | *-315.7* |  | *hydroxyl-carboxylate exchange n-C_8_ + EtOH 🡪 n-C_8_OH + C_2_* | *-12.3* |
| *n-octanol* | *-139.1* | *-176.6* |  |  |
| *n-hexanoate* | *-329.6* |  |  |  |

1. Roghair M, Hoogstad T, Strik DPBTB, Plugge CM, Timmers PHA, Weusthuis RA, et al. Controlling Ethanol Use in Chain Elongation by CO2 Loading Rate. Environmental Science & Technology. 2018;52(3):1496-505.

2. de Leeuw KD, Buisman CJN, Strik DPBTB. Branched Medium Chain Fatty Acids: Iso-Caproate Formation from Iso-Butyrate Broadens the Product Spectrum for Microbial Chain Elongation. Environmental Science & Technology. 2019;53(13):7704-13.

3. Angenent LT, Richter H, Buckel W, Spirito CM, Steinbusch KJJ, Plugge CM, et al. Chain Elongation with Reactor Microbiomes: Open-Culture Biotechnology To Produce Biochemicals. Environmental Science and Technology. 2016;50(6):2796-810.

4. Flamholz A, Noor E, Bar-Even A, Milo R. eQuilibrator—the biochemical thermodynamics calculator. Nucleic Acids Research. 2011;40(D1):D770-D5.

5. Noor E, Haraldsdóttir HS, Milo R, Fleming RMT. Consistent Estimation of Gibbs Energy Using Component Contributions. PLOS Computational Biology. 2013;9(7):e1003098.
